# Mechanistic insights into VSIR-mediated AXL regulation and gastric cancer proliferation

**DOI:** 10.1016/j.isci.2026.117580

**Published:** 2026-09-18

**Authors:** Jinshan Liu, Guoquan Huang, Yi Li, Yong Tan, Bo Qin, Changjiang Hao, Jilv Peng, Hongyang Zhu, Bitao Zhang, Yong Cheng, Kun Qian

**Affiliations:** 1 Department of Gastrointestinal Surgery, the First Affiliated Hospital of Chongqing Medical University, Chongqing 400016, China; 2 Hubei Key Laboratory for Translational Research in Traditional Chinese Medicine, Hubei Selenium and Human Health Institute, Department of Colorectal and Anal Surgery, The Central Hospital of Enshi Tujia and Miao Autonomous Prefecture, Hubei Minzu University, Enshi 445000, China; 3 Department of General Surgery, Chongqing Hospital of Jiangsu Province Hospital, The People’s Hospital of Qijiang District, Chongqing 401420, China; 4 Department of Hepatobiliary Surgery, The Central Hospital of Enshi Tujia and Miao Autonomous Prefecture, Enshi 445000, China; 5 State Key Laboratory of Biocatalysis and Enzyme Engineering, Hubei Key Laboratory of Industrial Biotechnology, School of Life Sciences, Hubei University, #368 Youyi Road, Wuhan 430062, P.R. China

**Keywords:** gastric cancer, VSIR, AXL, STAT3, HER2, combination therapy

## Abstract

Gastric cancer (GC) progression is frequently driven by complex tumor microenvironment signals. Here, our study identifies V-set immunoregulatory receptor (VSIR) as a key driver of GC progression via AXL regulation. High-throughput RNA sequencing demonstrated that VSIR overexpression potently induces AXL upregulation in response to lipopolysaccharide (LPS) stimulation. This finding was confirmed through both VSIR overexpression and knockdown experiments. Mechanistically, our data demonstrated that STAT3 directly contributes to AXL transcriptional upregulation. Crucially, VSIR not only enhances LPS induced STAT3 phosphorylation, thereby amplifying AXL transcription, but also interacts with the AXL protein to stabilize it, leading to sustained AXL signaling. Functionally, the VSIR-AXL axis drives GC proliferation; notably, AXL inhibition suppresses this tumorigenesis, while combined AXL and HER2 blockade synergistically abrogates tumor growth. These findings reveal a critical oncogenic pathway and provide a strong rationale for dual-targeted therapeutic strategies in GC management.

## Highlights

• VSIR promotes LPS-dependent AXL transcription through IL-6-STAT3 signaling
• VSIR binds AXL and protects it from ubiquitin-proteasome degradation
• AXL inhibition suppresses VSIR-driven gastric cancer growth and migration
• Combined AXL and HER2 blockade produces a potent antitumor effect in gastric cancer

## Graphical abstract

**Figure:**
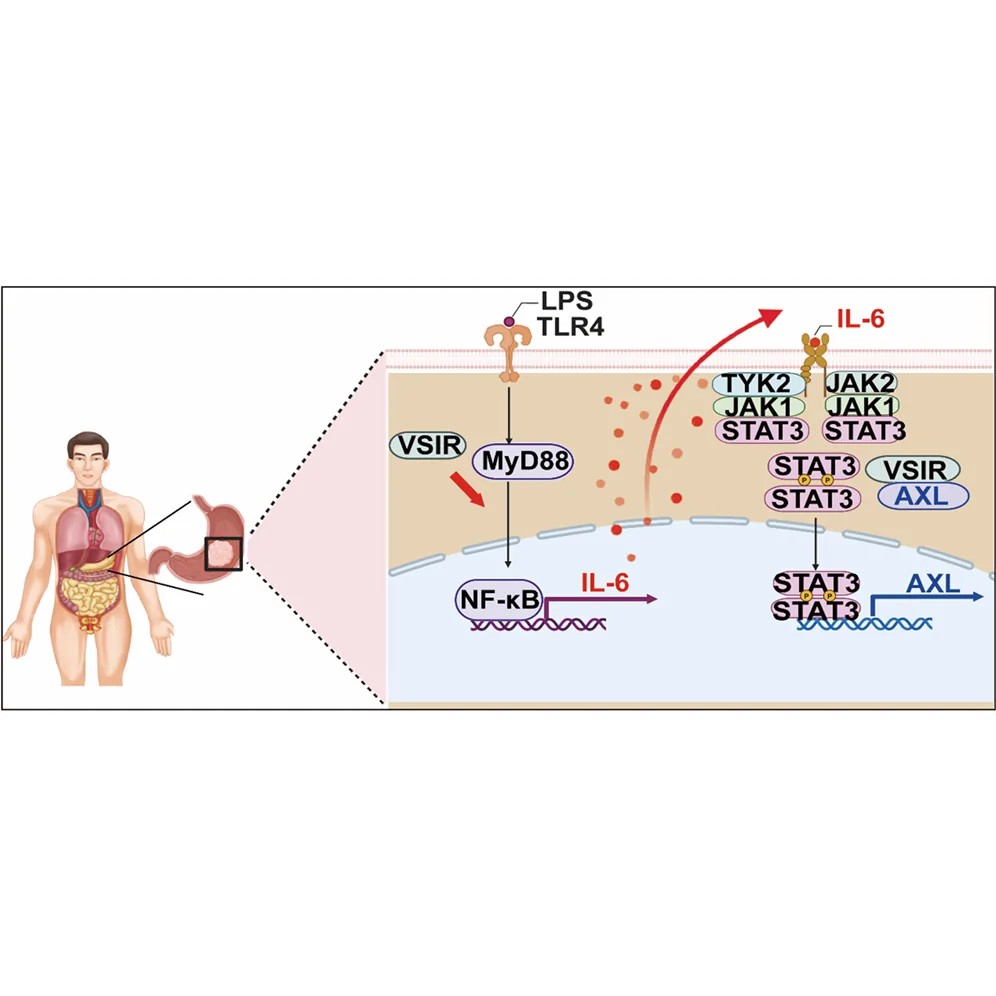

## Teaser

Molecular biology; Immunology; Cancer

## Introduction

Gastric cancer (GC) remains one of the most lethal malignancies worldwide, ranking as the fifth most commonly diagnosed malignancy worldwide and accounting for approximately 7.7% of all cancer-related deaths.^1^ A major etiological driver of this disease is chronic infection with *Helicobacter pylori* , a Gram-negative bacterium that colonizes the gastric mucosa in more than half of the global population.^2^ *H. pylori* infection induces sustained mucosal inflammation and epithelial injury, largely mediated by its lipopolysaccharide (LPS), which engages Toll-like receptor 4 (TLR4) signaling.^3^ Activation of the TLR4 axis promotes the production of pro-inflammatory cytokines, particularly interleukin 6 (IL-6), which subsequently triggers persistent STAT3 activation in gastric epithelial cells and immune compartments.^4^^,^^5^ This IL-6-STAT3 signaling cascade reinforces inflammatory circuits and enhances epithelial survival and proliferation, thereby establishing a microenvironment permissive for gastric tumorigenesis.^6^ Importantly, sustained LPS-driven inflammation not only disrupts gastric homeostasis but also acts as a critical driving force for tumor progression, highlighting the necessity to identify specific downstream mediators that are directly regulated by STAT3 to bridge chronic inflammation and malignant phenotypes. Concurrently, the advent of molecularly targeted therapies has shifted the treatment paradigm, particularly with the recognition of HER2 as a pivotal driver in a subset of GCs. Approximately 10%–20% of gastric adenocarcinomas overexpress HER2,^7^ and trastuzumab-based regimens have become the first-line standard.^8^ However, targeting a single oncogenic driver is often insufficient to completely halt tumor progression in complex microenvironments. This underscores the urgent need for novel combinatorial strategies that simultaneously target both canonical drivers and inflammation-associated adaptive pathways to achieve robust anti-tumor efficacy.

V-set immunoregulatory receptor (VSIR) is a type I transmembrane immune checkpoint molecule of the B7/CD28 family that plays a central role in regulating immune homeostasis. It was initially characterized for its potent immunosuppressive effects within the tumor microenvironment (TME), where it attenuates effector T cell activation, enhances regulatory T cell (Treg) expansion, and contributes to the suppressive functions of myeloid-derived suppressor cells (MDSCs) and tumor-associated macrophages.^9^^,^^10^ In addition to its expression on various immune cell subsets, recent studies have documented frequent upregulation of VSIR on tumor cells themselves in multiple cancer types, implicating tumor-intrinsic VSIR expression in promoting immune escape and shaping the immunosuppressive milieu.^11^ Notably, emerging evidence suggests that VSIR may have tumor cell-associated functions beyond classical immune regulation. In melanoma, tumor cell-specific VSIR expression has been documented and, in preclinical models, promotes tumor onset; in multiple cancer types, elevated VSIR expression has been correlated with aggressive clinical features and poor outcomes.^12^^,^^13^ In GC, elevated VSIR expression has been reported to correlate with advanced tumor stage, lymph node metastasis, and unfavorable clinical outcomes.^14^^,^^15^ Collectively, these findings raise the possibility that, in addition to its established role as an immune checkpoint, VSIR may participate in tumor progression through tumor-intrinsic mechanisms; however, the underlying signaling pathways and causal relationships remain to be fully elucidated.

AXL, a receptor tyrosine kinase of the TAM family (TYRO3/AXL/MERTK), has emerged as a critical regulator of tumor progression, metastatic dissemination, and therapeutic resistance in diverse cancers.^16^^,^^17^ Activation of AXL signaling promotes epithelial-mesenchymal transition (EMT) and invasive behavior in breast and other solid tumors^18^ and confers resistance to targeted therapies and chemotherapy in lung cancer,^19^^,^^20^ and modulates anti-tumor immune responses in ways that undermine checkpoint blockade efficacy.^21^ These attributes make AXL a compelling target for therapeutic intervention, with multiple preclinical and clinical strategies in development.^22^ Upon binding to its ligand GAS6, AXL activates downstream pathways such as PI3K/AKT, MAPK/ERK, and STAT3, which collectively enhance cell survival, proliferation, EMT, and immune evasion.^21^^,^^23^ In GC, AXL overexpression has been documented in both cell lines and patient specimens and is associated with aggressive phenotypes, including peritoneal dissemination and poor overall survival.^24^^,^^25^^,^^26^^,^^27^ Preclinical studies demonstrate that AXL knockdown or pharmacological inhibition suppresses GC cell growth and sensitizes tumors to chemotherapy and targeted agents.^21^^,^^28^^,^^29^ Although large-scale clinical trials targeting AXL in GC are still limited, early-phase studies with AXL inhibitors bemcentinib (BGB324) in solid tumors show promise, supporting further investigation of AXL as both a biomarker and a therapeutic target in GC subsets.

Despite the established oncogenic role of the IL-6-STAT3 axis, the specific downstream effectors that directly mediate its malignant potential in GC remain to be fully characterized. Therefore, a primary objective of this study was to screen for and identify the critical targets governed by this signaling pathway. Furthermore, while VSIR is well-recognized as an immune checkpoint,^10^^,^^30^ its potential involvement in intrinsic inflammatory regulation within tumor cells remains largely unexplored. We thus hypothesized that VSIR might orchestrate pro-tumorigenic programs by modulating these key signaling nodes. Finally, we aimed to evaluate the therapeutic potential of targeting this newly defined axis, specifically examining whether its combination with HER2 inhibition could yield superior antitumor efficacy. Here, we demonstrate that the combined targeting of AXL and HER2 effectively abrogates tumor proliferation and migration. Collectively, our work identifies the VSIR-AXL signaling axis as a novel therapeutic vulnerability and provides a strong rationale for co-targeting AXL and HER2 to synergistically enhance antitumor efficacy and improve clinical outcomes in GC.

## Results

### VSIR promotes AXL mRNA expression in an LPS-dependent manner

To evaluate the clinical relevance of AXL in GC, we retrieved publicly available data from The Cancer Genome Atlas Stomach Adenocarcinoma (TCGA-STAD) cohort through the GEPIA2 platform. As shown in Figure 1A, AXL expression levels increased progressively with tumor stage, being significantly higher in Stage IV compared to Stage I (Figure 1A). Additionally, Kaplan-Meier survival analysis based on TCGA-STAD data shows that patients with high AXL expression had significantly worse overall survival than those with low expression (Figure 1B). These publicly available data suggest a strong association between AXL upregulation, disease progression, and poor prognosis in GC. Consistent with our observation from the TCGA-STAD dataset that AXL is upregulated in advanced GC and associated with poor prognosis, we sought to investigate the regulatory mechanisms underlying AXL overexpression. Transcriptome sequencing revealed that *AXL* mRNA was markedly elevated under conditions of VSIR overexpression combined with LPS stimulation (Figure 1C). To validate this finding, we performed RT-qPCR in HGC-27 cells and confirmed that VSIR overexpression failed to induce *AXL* mRNA in the absence of LPS; however, upon LPS treatment, VSIR significantly enhanced AXL transcript levels (Figures 1D and 1F). Western blot analysis further corroborated these results at the protein level (Figure 1G). To determine whether VSIR is necessary for LPS-induced AXL expression, we designed siRNAs targeting VSIR and transfected them into HGC-27 cells prior to LPS stimulation. Knockdown of VSIR effectively abolished the LPS-mediated induction of AXL expression (Figures 1H–1J), indicating that VSIR is required for AXL upregulation in response to LPS. Collectively, these data demonstrate that VSIR promotes AXL expression specifically under inflammatory conditions mimicked by LPS, providing a potential mechanistic link between innate immune signaling and AXL-driven tumor progression in GC.

**Figure 1:**
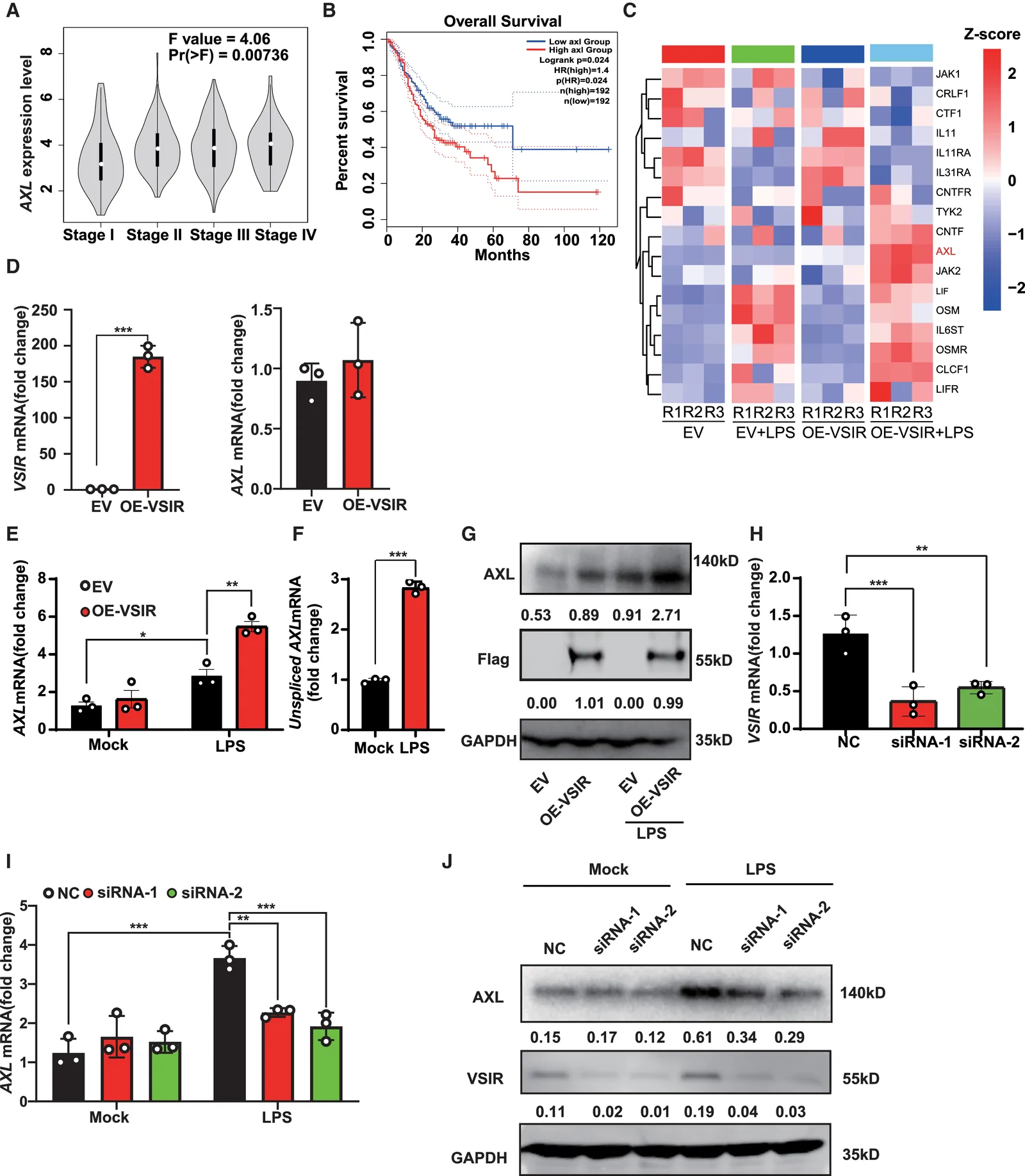
VSIR promotes *AXL* mRNA expression in an LPS-dependent manner (A) AXL expression levels across tumor stages (I–IV) in patients with stomach adenocarcinoma (STAD). Violin plots show distribution and median (black line). (B) Kaplan-Meier survival curves showing overall survival (OS) based on AXL expression levels. Patients were stratified into low and high AXL expression groups, with 192 patients in each group. The high AXL group showed significantly worse overall survival compared to the low AXL group (log rank *p* = 0.024; hazard ratio [HR] = 1.4). (C) Heatmap showing the expression levels of inflammatory-related genes in different experimental groups. Clustering was performed based on gene expression patterns. R1, R2, and R3 represent biological replicates. EV, empty vector control; OE-VSIR, overexpression of VSIR; and LPS, lipopolysaccharide stimulation. (D) The efficiency of VSIR overexpression and its effect on AXL mRNA expression were determined by RT-qPCR in the HGC-27 cell line. (E–G) AXL expression levels were analyzed by RT-qPCR and western blot, respectively, in an HGC-27 cell line stably overexpressing VSIR, with samples collected 8 h after LPS treatment. (H–J) VSIR was knocked down in HGC-27 cells using two independent siRNAs, and knockdown efficiency was confirmed by RT-qPCR (H). Following VSIR knockdown, cells were stimulated with LPS for 8 h, and AXL expression was assessed at both the mRNA level by RT-qPCR (I) and the protein level by western blot (J). Data are representative of three independent experiments (D–J). Mean ± SD, ∗*p* < 0.05; ∗∗*p* < 0.01; and ∗∗∗*p* < 0.001 indicate significant difference was assessed using two-tailed Student’s *t* test or two-way ANOVA.

### STAT3 directly binds to the AXL enhancer and drives its transcriptional activation

To identify potential transcriptional regulators of AXL, we performed computational prediction using multiple transcription factor-binding site databases: hTFtarget, CHEA, FIMO_JASPAR, and ENCODE. As shown in Figure 2A, a Venn diagram analysis revealed that two transcription factors—STAT3 and EGR1—were commonly predicted to bind to the regulatory regions of the *AXL* gene across all four datasets, suggesting their potential role in AXL expression regulation (Figure 2A). To further validate the functional relevance of these candidates, we examined chromatin accessibility and histone modification profiles at the *AXL* locus using the Cistrome Database (Cistrome DB). We observed strong enrichment of STAT3-binding signals within an enhancer region upstream of the *AXL* gene, coinciding with active histone marks H3K27ac and H3K4me1 (Figure 2B). In contrast, no such regulatory signatures were detected for IGF1R. These data indicate that STAT3 is likely bound to a functional enhancer element near AXL and may play a direct role in its transcriptional regulation. Based on these findings, we selected STAT3 as a candidate regulator for further investigation. To experimentally validate the bioinformatic prediction that STAT3 regulates AXL expression, we first performed chromatin immunoprecipitation followed by qPCR (ChIP-qPCR) in HGC-27 cells. Using primers spanning the putative enhancer region upstream of the *AXL* gene identified in Cistrome DB, we detected significant enrichment of STAT3-binding at this locus compared with control IgG (Figure 2C), confirming a direct physical interaction between STAT3 and the AXL regulatory region. Next, we cloned the enhancer fragment containing the predicted STAT3-binding motif into a luciferase reporter vector. To confirm the specificity of this binding, we generated a mutant construct with site-directed mutations in the STAT3-binding motif, as predicted by the JASPAR database. Co-transfection of the wild-type reporter with a STAT3 expression plasmid markedly increased luciferase activity (Figure 2D). Conversely, this STAT3-induced activation was significantly attenuated when the mutated reporter construct was used (Figure 2D), confirming that STAT3 directly regulates transcription through this specific motif. Finally, to assess the functional dependency of AXL on STAT3, we knocked down STAT3 using siRNA in HGC-27 cells. Both RT-qPCR and western blot analyses revealed that STAT3 depletion significantly reduced *AXL* mRNA and protein levels (Figures 2E and 2F). Collectively, these data establish STAT3 as a transcription factor that directly binds to an enhancer element upstream of AXL and is essential for its basal and inducible expression in GC cells.

**Figure 2:**
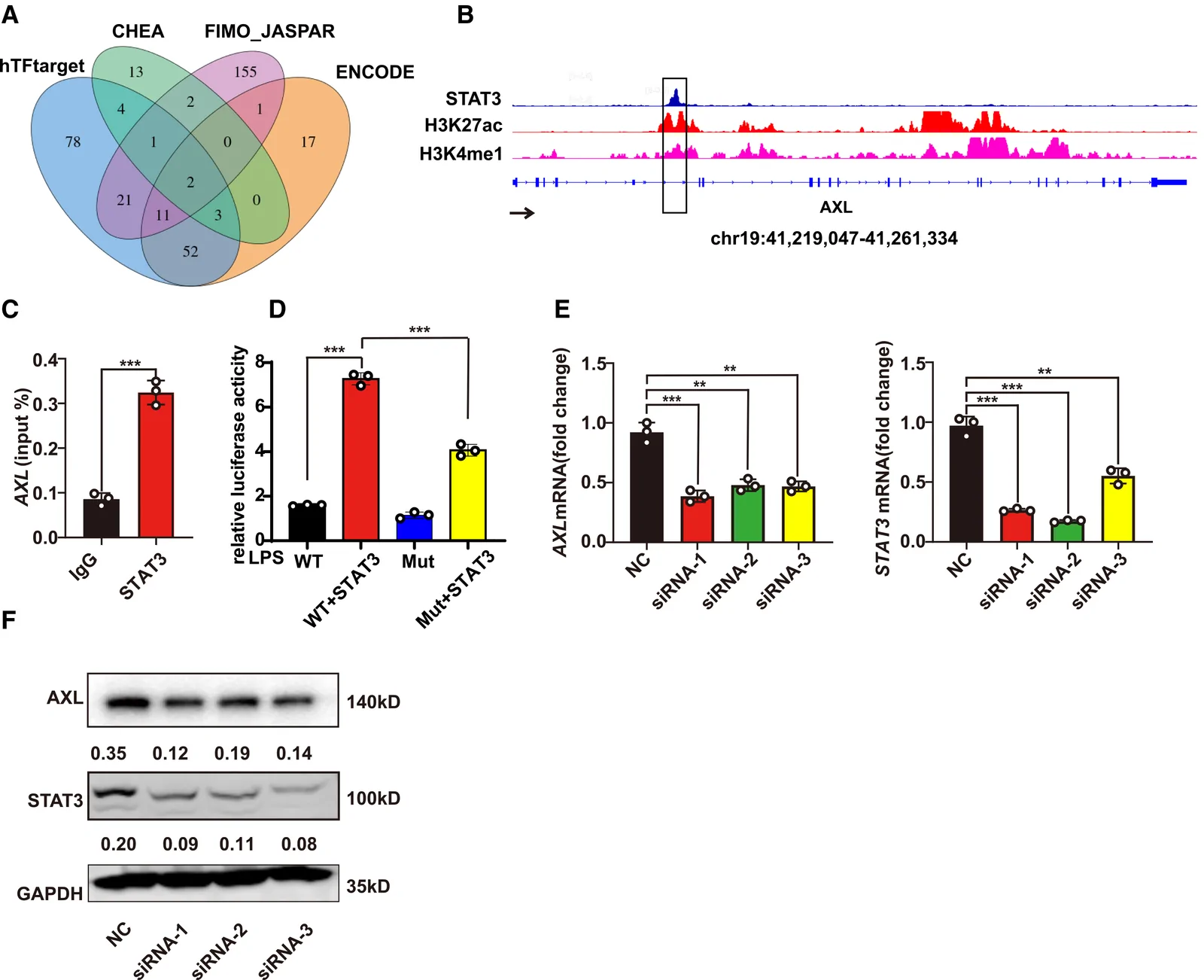
STAT3 directly binds to the AXL enhancer and drives its transcriptional activation (A) Venn diagram illustrating the overlap of predicted AXL regulatory elements identified by four transcription factor-binding site prediction tools: hTFtarget, CHEA, FIMO_JASPAR, and ENCODE. The numbers in each region represent the count of overlapping regulatory sites predicted by one or more tools. Notably, within the central overlap shared by all four databases, two key genes-EGR1 and STAT3 are identified, suggesting their potential roles in the regulation of AXL. (B) Chromatin immunoprecipitation sequencing (ChIP-seq) signal profiles of STAT3-binding (GSE79707), H3K27ac (histone H3 lysine 27 acetylation), and H3K4me1 (histone H3 lysine 4 monomethylation) across the genomic locus encompassing the *AXL* gene (GSE29118). Blue peaks indicate regions enriched for STAT3, red peaks represent H3K27ac enrichment, and pink peaks denote H3K4me1 distribution. Black arrows indicate the direction of gene transcription, and blue vertical bars below mark exon positions. (C) ChIP-qPCR analysis showing the enrichment of STAT3 at the *AXL* gene enhancer region. (D) Dual-luciferase reporter assays were performed to assess the transcriptional activity of the AXL enhancer region. Cells were co-transfected with luciferase reporter plasmids containing either the wild-type (WT) or mutant (mut) AXL enhancer sequence, along with either an empty vector (EV) or a STAT3 expression vector. (E and F) Concurrent RT-qPCR and western blot analyses show that silencing of STAT3 leads to a significant decrease in both *AXL* mRNA and protein expression compared to the negative control (NC) in HGC-27 cells. Data are representative of three independent experiments (C–F). Mean ± SD; ∗*p* < 0.05; ∗∗*p* < 0.01; and ∗∗∗*p* < 0.001 indicate significant difference was assessed using two-tailed Student’s *t* test.

### STAT3 is essential for LPS-induced AXL upregulation

To investigate the physiological relevance of STAT3 in mediating LPS-induced AXL expression, we isolated bone marrow-derived macrophages (BMDMs) from wild-type (WT) mice and transfected them with either control siRNA (si-NC) or specific siRNA targeting STAT3 (si-STAT3). Following transfection, the cells were stimulated with LPS. Western blot analysis revealed that while LPS treatment significantly increased AXL protein levels in the si-NC group, this induction was completely abolished in the si-STAT3 BMDMs (Figure 3A), indicating that STAT3 is required for LPS-mediated AXL upregulation in primary myeloid cells. To confirm these findings in a human GC cell model, we performed siRNA-mediated knockdown of STAT3 in HGC-27 cells. Consistent with the results, LPS-induced AXL expression was markedly reduced upon STAT3 depletion (Figure 3B), under both basal and LPS-stimulated conditions. Together, these data demonstrate that STAT3 is a critical transcriptional mediator of LPS-induced AXL expression in both myeloid cells and GC cells.

**Figure 3:**
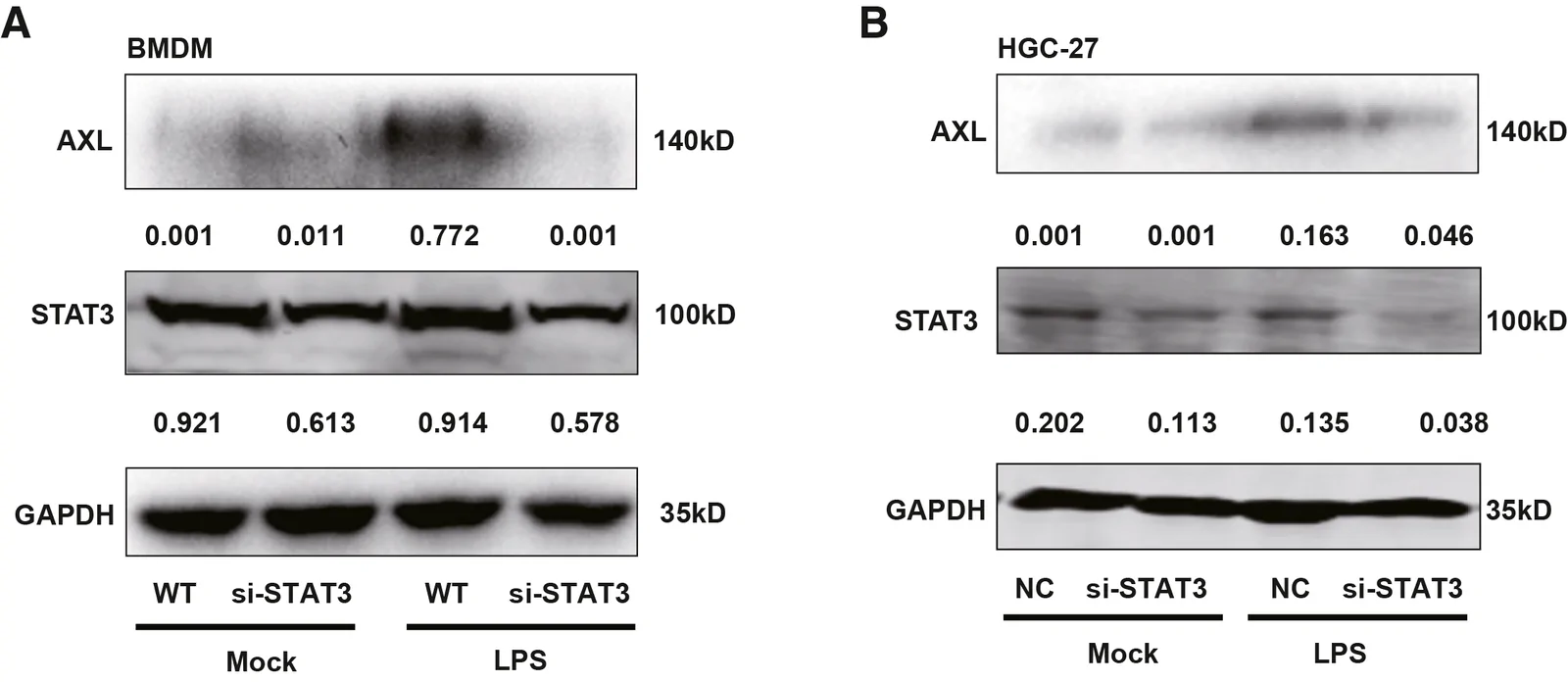
STAT3 is essential for LPS-induced AXL upregulation (A) Western blot analysis of AXL protein expression in BMDM after STAT3 knockdown (si-STAT3, 48 h) followed by LPS stimulation. (B) Western blot analysis of AXL protein expression in HGC cells after STAT3 knockdown (si-STAT3, 48 h) followed by LPS stimulation. Data are representative of three independent experiments.

### VSIR promotes AXL mRNA expression by enhancing IL-6 production and STAT3 activation under LPS stimulation

In the presence of LPS stimulation, overexpression of VSIR markedly increased IL-6 expression at the mRNA level, as evidenced by both heatmap analysis and RT-qPCR (Figures 4A and 4B). Concomitantly, VSIR overexpression induced phosphorylation of STAT3, indicating activation of the STAT3 signaling pathway, as confirmed by western blotting (Figure 4C). Notably, no significant differences were observed in the phosphorylation levels of p65 or JAK1 (Figure 4C), suggesting the specificity of this pathway activation. To investigate whether this VSIR-induced upregulation is TLR4-dependent, we knocked down TLR4 in HGC-27 cells. The results showed that TLR4 depletion abrogated the VSIR-induced enhancement of *IL6* mRNA levels, demonstrating that VSIR acts downstream of TLR4 (Figure 4D). Collectively, these data demonstrate that VSIR upregulates *AXL* mRNA expression through the potentiation of the TLR4/IL-6/STAT3 signaling cascade.

**Figure 4:**
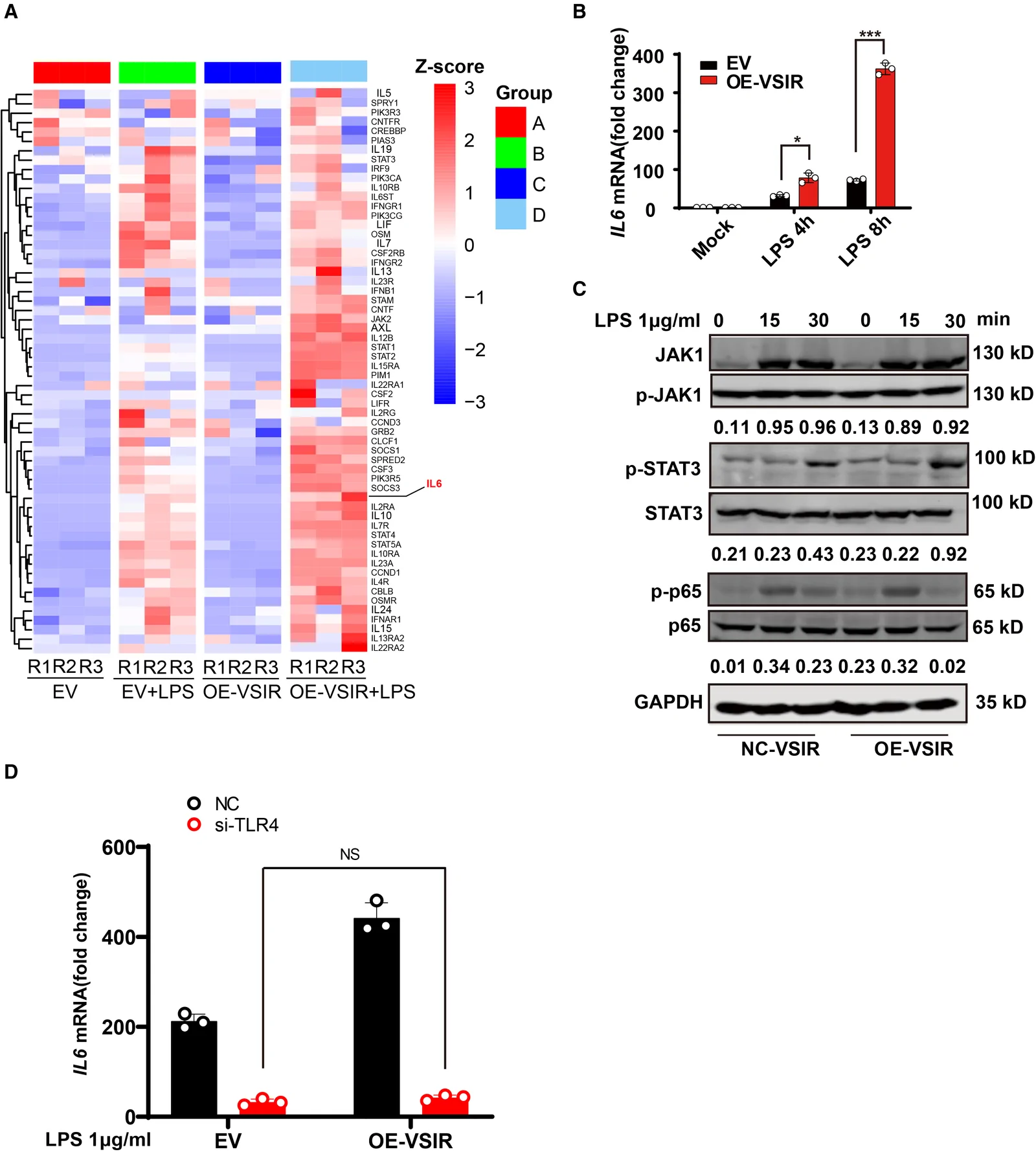
VSIR promotes *AXL* mRNA expression by enhancing IL-6 production and STAT3 activation under LPS stimulation (A) Heatmap showing the expression levels of inflammatory-related genes in different experimental groups. Clustering was performed based on gene expression patterns. R1, R2, and R3 represent biological replicates. EV: empty vector control; OE-VSIR: overexpression of VSIR; and LPS: lipopolysaccharide stimulation. (B and C) In HGC-27 cells stably overexpressing VSIR (OE-VSIR) or control vector (EV), *IL6* mRNA expression was assessed by RT-qPCR at 4 and 8 h post-LPS stimulation (B), and total phosphorylated levels of STAT3, p65, and JAK1 were analyzed by western blot at the indicated time points (C). GAPDH served as the loading control. (D) HGC-27 cells stably expressing empty vector (EV) or VSIR (OE-VSIR) were transfected with TLR4-targeting siRNA (si-TLR4) or control siRNA. Following LPS stimulation, *IL6* mRNA levels were quantified by RT-qPCR. Data are representative of three independent experiments (B and C). Mean ± SD; ∗*p* < 0.05, ∗∗*p* < 0.01, and ∗∗∗*p* < 0.001 indicate significant difference was assessed using two-way ANOVA.

### VSIR promotes AXL protein stability

Our previous data demonstrated that VSIR overexpression enhances the LPS-induced IL-6-STAT3 signaling pathway and upregulates AXL expression. Here, we further show that VSIR interacts with AXL, leading to increased stability of AXL protein levels. Western blot analysis revealed a dose-dependent increase in AXL protein expression upon VSIR-HA overexpression (Figure 5A), while co-immunoprecipitation experiments confirmed the interaction between VSIR and AXL (Figure 5B). To determine whether this upregulation results from enhanced protein stability, we performed cycloheximide (CHX) chase assays, which showed that VSIR overexpression significantly prolonged the half-life of the AXL protein (Figure 5C). Consistent with this, treatment with the proteasome inhibitor MG132 abolished the difference in AXL protein levels between NC and si-VSIR cells. This result suggests that VSIR prevents AXL degradation via the ubiquitin-proteasome pathway (Figure 5D). Indeed, ubiquitination assays demonstrated that VSIR overexpression significantly attenuated the polyubiquitination levels of endogenous AXL (Figure 5E). Furthermore, immunofluorescence staining confirmed the colocalization of VSIR and AXL in HGC-27 and MKN-45 cells (Figure 5F). These findings suggest that VSIR not only modulates AXL expression transcriptionally but also stabilizes AXL protein via interaction, thereby amplifying downstream inflammatory signaling pathways such as IL-6-STAT3.

**Figure 5:**
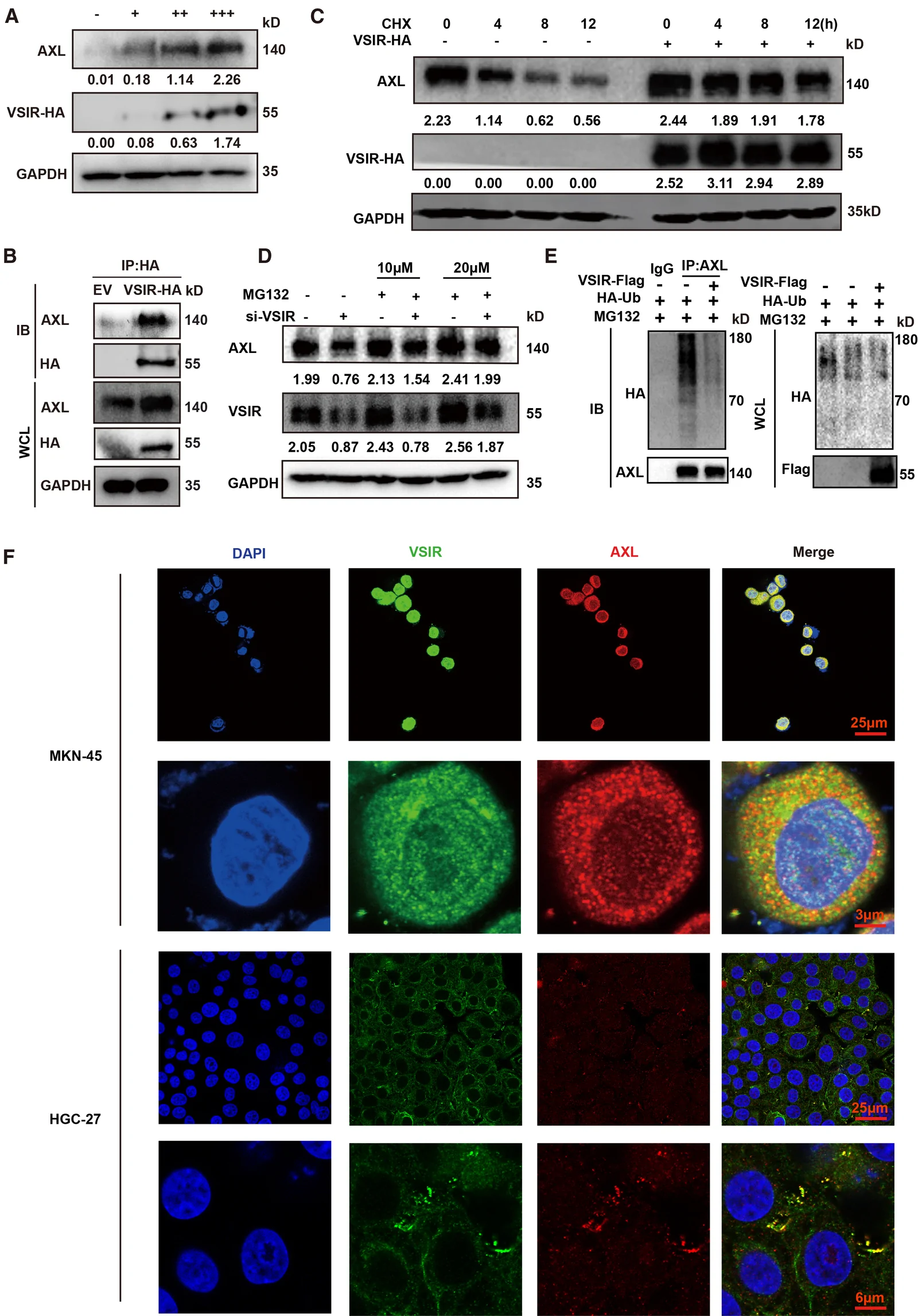
VSIR promotes AXL protein stability (A) HGC-27 cells were transfected with increasing amounts (0, 500, 1,000, and 2,000 ng) of VSIR plasmid, and AXL protein expression was analyzed by western blot. (B) Following VSIR overexpression in HGC-27 cells, co-immunoprecipitation (coIP) was performed, and AXL was detected by western blot to assess potential interaction. (C) HGC-27 cells were transfected with VSIR-HA overexpression plasmid or control vector for 24 h, followed by treatment with cycloheximide (CHX) for 0, 4, 8, and 12 h. AXL protein levels were then assessed by western blot. (D) HGC-27 cells were transfected with VSIR-targeting siRNA for 48 h, followed by treatment with MG132 (8 h). AXL protein levels were evaluated by western blot. (E) HGC-27 cells were co-transfected with VSIR-Flag/EV and Ub-HA for 24 h. Cell lysates were subjected to immunoprecipitation (IP) using an anti-AXL antibody, and the ubiquitination level of AXL was detected by western blot using an anti-HA antibody. (F) Immunofluorescence staining to examine the subcellular co-localization of VSIR and AXL in MKN-45 cells (nuclei were counterstained with DAPI. Scale bars: 25 μm; local area magnification: scale bars: 3 μm) and HGC-27 cells (nuclei were counterstained with DAPI. Scale bars: 25 μm; local area magnification: scale bars: 6 μm). Data are representative of three independent experiments (A–E).

### Enrichment of VSIR^+^/AXL^+^/HER2^+^ triple-positive cells in HER2^+^ tumors

To validate the clinical relevance of our mechanistic findings, we first performed immunofluorescence staining to examine the expression patterns of VSIR, AXL, and p-STAT3 in GC tissues. Consistent with our *in vitro* data demonstrating that VSIR promotes IL-6-mediated p-STAT3 activation to upregulate and stabilize AXL, we observed a significant positive correlation among VSIR, AXL, and p-STAT3 levels in patient samples (Figures 6A and 6B). Building on this, we further investigated the clinical relevance of this axis by performing multiplex immunofluorescence staining on a cohort comprising HER2-positive treatment-resistant (HER2^+^/TR), HER2-positive treatment-naive (HER2^+^/TN), HER2-negative (HER2^-^), and normal gastric tissues. Our results revealed significantly elevated expression levels of both VSIR and AXL in HER2^+^ tumors compared with normal mucosa and HER2^-^ samples (Figures 6C–6F). Quantitative analysis confirmed that the percentage of VSIR^+^/AXL^+^/HER2^+^ triple-positive cells was markedly higher in HER2^+^ resistant tumors than in other groups (Figure 6G). This distinct co-expression pattern indicates that the VSIR-driven p-STAT3/AXL signaling is a common feature of HER2^+^ GC, rather than a specific marker for acquired resistance. These findings support our central hypothesis that HER2^+^ tumors rely on a dual mechanism: HER2-driven proliferation and VSIR-driven AXL-mediated survival. Consequently, concurrent targeting of VSIR and AXL may offer a promising therapeutic strategy to overcome the compensatory pathways that limit the efficacy of HER2-targeted monotherapy.

**Figure 6:**
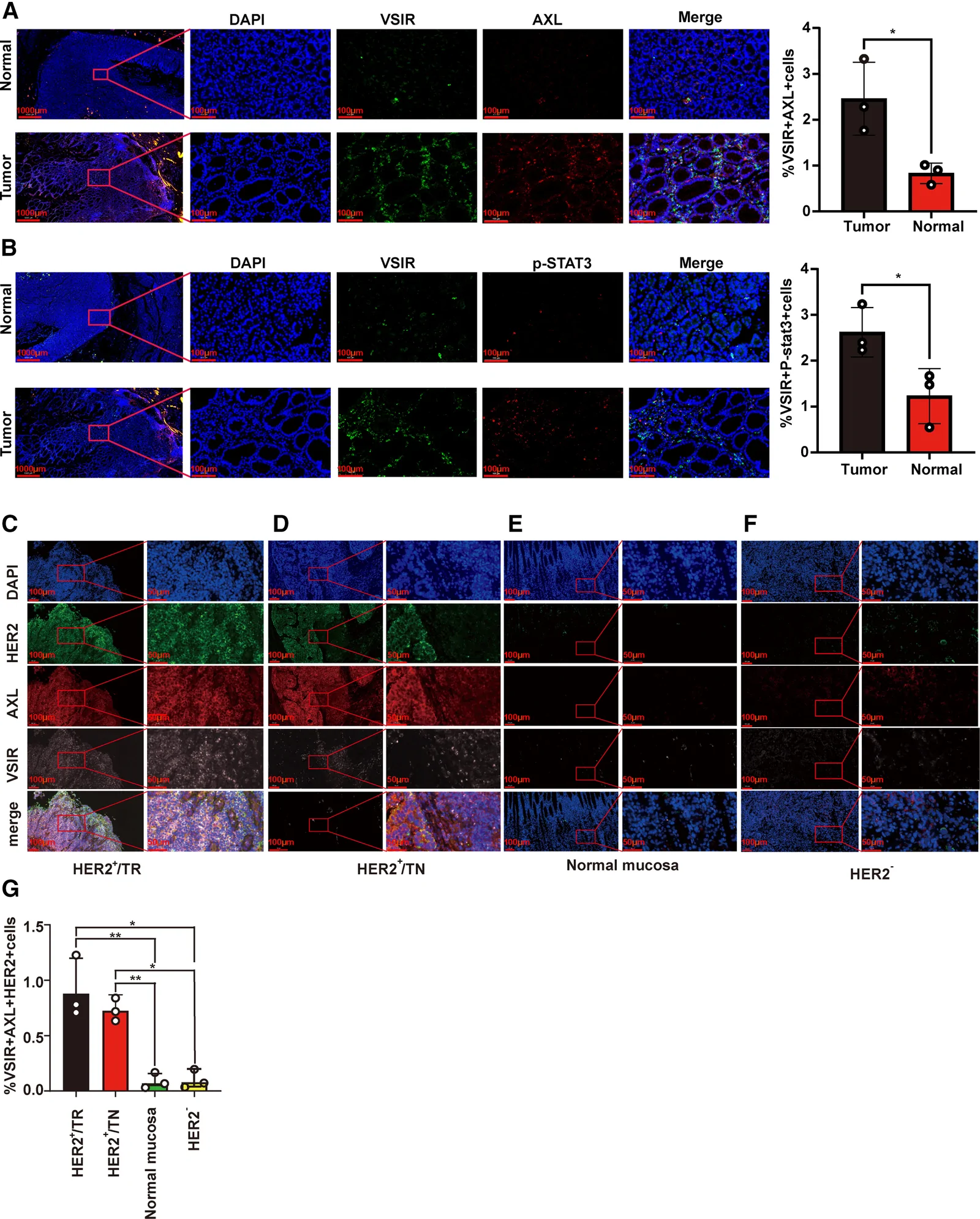
Enrichment of VSIR^+^/AXL^+^/HER2^+^ triple-positive cells in HER2^+^ tumors (A) The expression of VSIR and AXL was detected by immune multilabel experiment in human gastric tumor tissues and normal gastric mucosa tissues. The halo analysis software was used to analyze the immunohistochemical sections of panoramic scanning, and the positive rate (positive rate was equal to the number of positive cells over the total number of tissue cells) was obtained, bar graphs represent the positive rate of VSIR and AXL after quantification in sections, and the difference was statistically significant (Nuclei were counterstained with DAPI. Scale bars: 1,000 μm; local area magnification: scale bars: 100 μm). (B) The expression of VSIR and p-STAT3 was detected by immune multilabel experiments in human gastric tumor tissues and normal gastric mucosa tissues. The halo analysis software was used to analyze the immunohistochemical sections of panoramic scanning, and the positive rate (positive rate was equal to the number of positive cells over the total number of tissue cells) was obtained, bar graphs represent the positive rate of VSIR and p-STAT3 after quantification in sections, and the difference was statistically significant (nuclei were counterstained with DAPI. Scale bars: 1,000 μm; local area magnification: scale bars: 100 μm). (C–F) Multiplex immunofluorescence staining of VSIR, HER2, and AXL in HER2^+^ gastric cancer patients with resistance to HER2-targeted therapy (C), treatment-naïve gastric cancer patients (D), normal gastric mucosa (E), and HER2^-^ gastric cancer patients (F) (nuclei were counterstained with DAPI. Scale bars: 100 μm; local area magnification: scale bars: 50 μm). (G) The halo analysis software was used to analyze the immunohistochemical sections of panoramic scanning, and the positive rate (positive rate was equal to the number of positive cells over the total number of tissue cells) was obtained. Bar graphs represent the positive rate of VSIR/AXL/HER2 after quantification in sections of (C)–(F). Data are representative of three independent experiments. Mean ± SD; ∗*p* < 0.05 and ∗∗*p*< 0.01 indicate significant difference was assessed using two-tailed Student’s *t* test.

### AXL is required for VSIR-mediated tumor growth and metastatic potential

To evaluate the functional role of VSIR in GC progression *in vivo*, we established a subcutaneous xenograft tumor model in nude mice using HGC-27 GC cells stably overexpressing VSIR (OE-VSIR) or empty vector control (NC). Tumor-bearing mice were randomized into five treatment groups (*n* = 5 per group): (1) negative control (NC); (2) OE-VSIR; (3) OE-VSIR + HER2 inhibitor; (4) OE-VSIR + AXL inhibitor; and (5) OE-VSIR + dual HER2/AXL inhibitors. After 30 days of treatment, tumors were harvested for morphological and molecular analysis. The results showed that OE-VSIR significantly promoted tumor growth compared with the NC group, as evidenced by increased tumor volume and weight (Figures 7A–7C). Notably, administration of the AXL inhibitor alone markedly suppressed VSIR-induced tumor growth, reducing both tumor size and weight to levels comparable to or below those of the control group. In contrast, HER2 inhibition alone provided only modest suppression of tumor growth. Strikingly, combined blockade of HER2 and AXL yielded the most potent anti-tumor effect, nearly abolishing the pro-tumorigenic activity of VSIR (Figures 7A–7C). To assess proliferation *in vivo*, Ki-67 expression was evaluated by immunohistochemistry (IHC) in tumor sections (Figure 7D). Consistent with the tumor growth data, Ki-67 staining intensity was highest in the OE-VSIR group and substantially reduced upon AXL inhibition. Quantitative analysis of Ki-67 immunoreactivity using HALO software confirmed these observations, showing a significant decrease in integrated optical density (IOD) in the AXL inhibitor and dual-inhibitor groups (Figure 7D). To further validate that AXL acts as a critical downstream effector of VSIR, we performed rescue experiments *in vitro* using VSIR-knockdown MKN-45 cells. Wound healing and Transwell assays demonstrated that while VSIR knockdown significantly impaired cell migration and invasion, these metastatic capabilities were substantially restored upon AXL overexpression (Figures 7E and 7F). In summary, VSIR enhances AXL expression by potentiating LPS-induced IL-6/STAT3 signaling and interacts with AXL to stabilize its protein levels, thereby promoting GC cell proliferation and tumor progression (Figure 7G).

**Figure 7:**
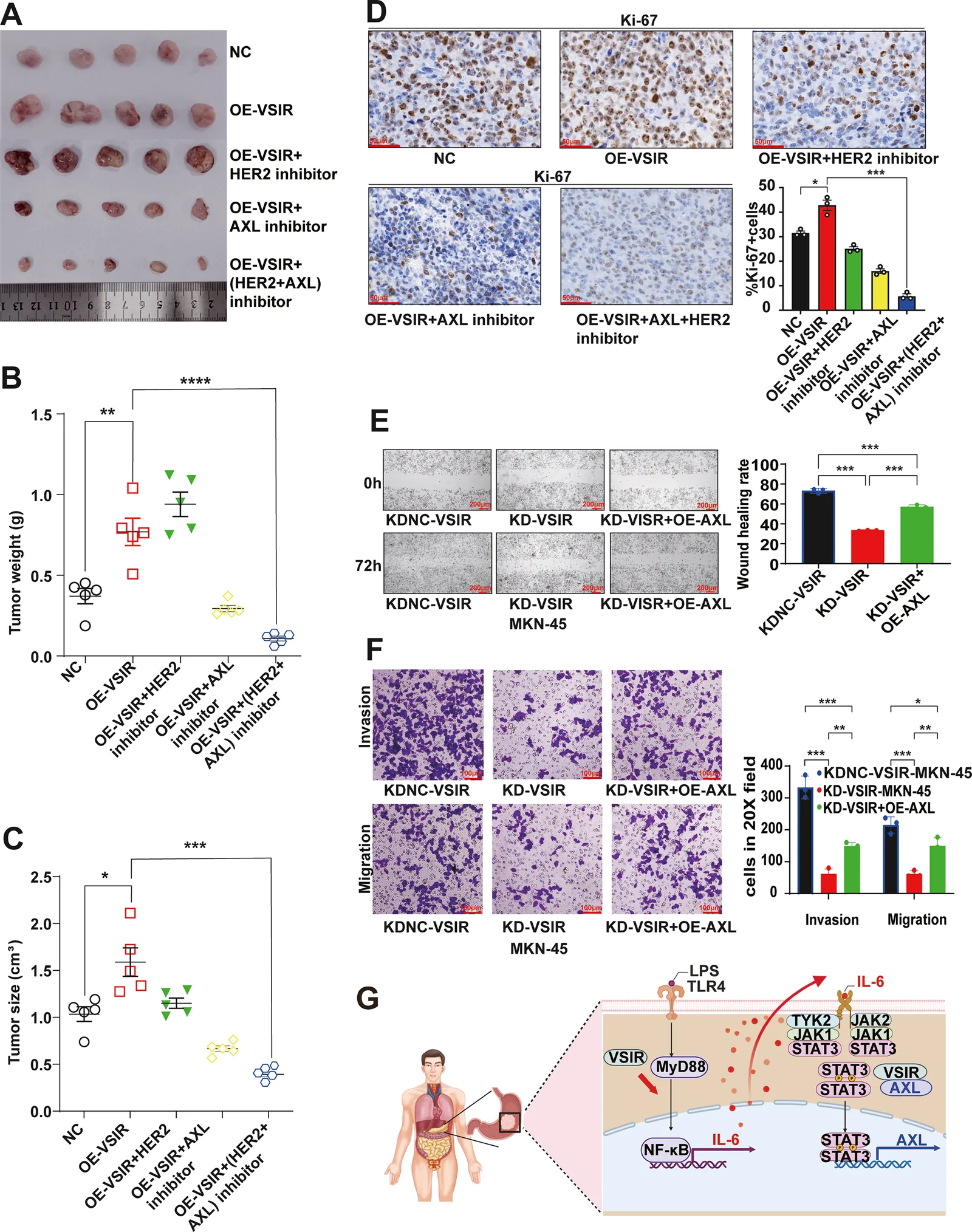
AXL is required for VSIR-mediated tumor growth and metastatic potential (A–C) A subcutaneous xenograft tumor model was established in nude mice using HGC-27 gastric cancer cells. Tumor-bearing mice were randomly assigned to five groups (*n* = 5 per group): (1) negative control (NC); (2) OE-VSIR; (3) OE-VSIR + HER2 inhibitor; (4) OE-VSIR + AXL inhibitor; and (5) OE-VSIR + HER2 + AXL inhibitors. After 30 days of treatment, tumor morphology (A), tumor weight (B), and tumor volume (C) were evaluated. (D) Ki-67 protein expression in tumor tissues from the five groups described in (A) was assessed by immunohistochemistry (IHC). Scale bars: 50 μm. The integrated optical density (IOD) of Ki-67 staining in tumor sections was quantified using HALO image analysis software. (E) Wound healing assay of MKN-45 gastric cancer cells in KDNC-VSIR, KD-VSIR, and KD-VSIR+OE-AXL groups. Micrographs captured at 0 and 72 h. Histograms show quantified scratch closure analyzed by ImageJ and plotted with GraphPad Prism. Scale bars as indicated; scale bars: 200 μm. (F) Transwell migration and invasion assays of MKN-45 gastric cancer cells across KDNC-VSIR, KD-VSIR, and KD-VSIR+OE-AXL groups. Histograms show quantitative cell counts analyzed by ImageJ and plotted with GraphPad Prism. Scale bars as indicated; scale bars: 100 μm. (G) Working model illustrating that VSIR promotes LPS-induced proliferation of GC cells by upregulating AXL expression, thereby facilitating tumor progression. Data are representative of three independent experiments (A–E). Mean ± SD; ∗*p* < 0.05; ∗∗*p* < 0.01; and ∗∗∗*p* < 0.001 indicate significant difference was assessed using two-tailed Student’s *t* test.

## Discussion

Our study establishes VSIR as a critical promoter of GC progression, functioning through a dual mechanism that couples transcriptional upregulation and post-translational stabilization of AXL. While VSIR has long been recognized as an immune checkpoint molecule expressed on myeloid and T cells, our findings reveal a tumor-intrinsic role for VSIR that directly fuels cancer progression-expanding its functional repertoire beyond immunosuppression. This is particularly significant in the context of GC, a disease characterized by profound molecular heterogeneity and the limited efficacy of current HER2-targeted therapies due to compensatory survival pathways.

Crucially, our data clarify the causal link between the inflammatory microenvironment and oncogenic signaling. We demonstrate that the LPS/IL-6/STAT3 pathway acts as an upstream trigger for AXL transcription, establishing the necessary conditions for AXL overexpression. However, transcription alone is insufficient for sustained oncogenic signaling. Here, VSIR functions as a critical downstream amplifier. By interacting with the AXL protein to prevent its degradation, VSIR stabilizes and prolongs AXL signaling. This coordinated regulation ensures robust AXL expression at both mRNA and protein levels. Importantly, our functional assays confirm that VSIR is required for this process, as VSIR knockdown effectively abolishes the LPS-induced AXL upregulation and tumorigenic phenotype.

From a therapeutic perspective, pharmacological inhibition of AXL not only abrogates VSIR-driven tumorigenesis but also exerts synergistic anti-tumor effects when combined with HER2 blockade. This suggests a rational combinatorial strategy for patients who fail current HER2-targeted therapies. While recent clinical efforts have focused on targets such as CLDN18.2 and FGFR2, AXL remains underexplored in GC despite its established roles in EMT and therapy resistance in other solid tumors.^16^^,^^31^^,^^32^ By elucidating how VSIR stabilizes AXL within an inflammatory context, our findings highlight the VSIR-AXL axis as a druggable target without overgeneralizing the role of inflammation.

Given the well-documented role of AXL in shaping an immunosuppressive tumor microenvironment—through mechanisms such as PD-L1 upregulation, recruitment of MDSCs, and polarization of macrophages toward an M2-like phenotype—our findings suggest that VSIR-mediated AXL activation may indirectly influence the tumor immune microenvironment. However, direct evidence for this link requires further validation. Notably, by linking inflammatory cues directly to oncogenic signaling, VSIR is implicated as a potential regulator in GC pathogenesis.

In conclusion, we uncover a non-immunological, pro-tumorigenic function of VSIR that operates through coordinated transcriptional and post-translational control of AXL. Targeting the VSIR-AXL axis, particularly with AXL inhibitors in combination with HER2-directed therapy, represents a promising avenue to overcome the compensatory pathways limiting the efficacy of HER2-targeted monotherapy and improve outcomes in GC, especially in aggressive, inflammation-associated subtypes. Future studies should evaluate AXL/HER2 co-inhibition in clinical trials to validate this dual-targeting strategy.

### Limitations of the study

While our work elucidates the critical role of the VSIR-AXL axis in GC progression, several mechanistic aspects remain to be fully elucidated. Firstly, although we demonstrate that VSIR enhances STAT3 phosphorylation, the precise mechanism by which VSIR facilitates the activation of the IL-6-STAT3 signaling pathway has not been thoroughly investigated. Secondly, while our data indicate that VSIR interacts with AXL to stabilize the protein, the exact molecular basis underlying this stabilization effect requires further in-depth exploration. Thirdly, because the *in vivo* experiments used only female mice, sex-dependent effects were not evaluated; this limits the generalizability of the animal findings across sexes.

## Resource availability

### Lead contact

Requests for further information and resources should be directed to and will be fulfilled by the lead contact, Kun Qian (hxjsqk@hospital.cqmu.edu.cn).

### Materials availability

All reagents generated in this study are available from the lead contact without restriction.

### Data and code availability

• The VSIR transcriptome sequencing data generated in this study are available at Zenodo: https://doi.org/10.5281/zenodo.19704242 (record identifier: 19704243).
• This paper does not report original code.
• Any additional information required to re-analyse the data reported in this paper is available from the lead contact upon request.

## Acknowledgments

We thank the Department of Pathology, First Affiliated Hospital of Chongqing Medical University, for their strong support and collaboration. This study was funded by 10.13039/501100005230Natural Science Foundation of Chongqing, China (no. CSTB2024NSCQ-MSX0098), Enshi Prefecture “Sailing Special Project” (no. D20250014), and Qian De Clinical Research Special Project (no. 2025QDLYZX-03).

## Author contributions

J.L., G.H., Y.C., and K.Q. were responsible for research design, specimen collection, and clinical data statistical analysis. J.L., G.H., Y.L., Y.T., B.Q., C.H., J.P., and B.Z. were responsible for conducting the experiments, data analysis, and mapping. J.L., G.H., and Y.L. wrote the article. J.L., G.H., B.Z, Y.C., and K.Q. were responsible for revising, polishing, submitting, and reworking this article.

## Declaration of interests

The authors declare no competing interests.

## STAR★Methods

### Key resources table

**Table:** 

REAGENT or RESOURCE	SOURCE	IDENTIFIER
**Antibodies**		

VSIR	Cell Signaling	RRID: AB_2799474
AXL	Cell Signaling	RRID: AB_11217435
STAT3	Cell Signaling	RRID: AB_2629499
GAPDH	Cell Signaling	RRID: AB_10622025
HA	Cell Signaling	RRID: AB_1549585
HER2	Fuzhou Maixin Biotechnology	RMA-1022
Ki-67	HUABIO	RRID: AB_3072239

**Deposited data**		

VSIR transcriptome sequencing dataset	National Genomics Data Center (NGDC)	PRJCA063405; https://ngdc.cncb.ac.cn/search/specific?db=bioproject&q=PRJCA063405

**Experimental models: Cell lines**		

HEK293T	American Type Culture Collection (ATCC)	N/A
HGC-27	Wuhan Punosai Life Technology Co. LTD (Wu han, China)	N/A
MKN-45	Wuhan Punosai Life Technology Co. LTD (Wu han, China)	N/A

**Experimental models: Organisms/strains**		

BALB/c nude mice	Wuhan Myhalic Biotechnology Co., Ltd., China	N/A

**Software and algorithms**		

ImageJ	NIH	https://imagej.nih.gov/ij/; RRID: SCR_003070
Prism (Graphpad)	GraphPad software Version 10.1.2	https://www.graphpad.com/
Adobe Illustrator	Adobe Illustrator (2023)	https://www.adobe.com/ creativecloud.html
HALO image analysis software	Indica Labs	Version: Halo 3.4.2986 , Identifier: Lndica Labs.

### Experimental model and study participant details

#### Patient, tissue and ethical statement

We collected gastric cancer patients who underwent surgery and received targeted therapy in the Department of Gastrointestinal Surgery of the First Affiliated Hospital of Chongqing Medical University. The collected tissues included gastric cancer tissues and adjacent normal tissues. All samples were approved by the Ethics Committee of the First Affiliated Hospital of Chongqing Medical University (Chongqing, China; Ethics Approval Number: K2023-100, 2026-0273-01).

Written informed consent was obtained from all patients prior to sample collection. The study included a total of 12 patients (9 males and 3 females, aged 55-78 years) for immune multi-label immunofluorescence experiments. Patient demographic details including age, sex, ethnicity, TNM stage, and HER2 status are provided in Tables S1 and S2. All patients were of Han nationality ethnicity.

#### Animal experiments

All animal procedures were approved by the Institutional Animal Care and Use Committee of Enshi Tujia and Miao Autonomous Prefecture Central Hospital (Ethical Approval No.:202412001) and conducted in accordance with the institutional Standard Operating Procedures (SOP). Four- to five-week-old female BALB/c nude mice (Wuhan Myhalic Biotechnology Co., Ltd., China) were used to establish subcutaneous gastric cancer xenografts. According to literature reports and reagent instructions, we confirmed that the animal model used was suitable for AXL and HER2-targeted inhibition.^33^

HGC-27 and HGC-27-VSIR-overexpressing stable cell lines were cultured, detached with trypsin, and centrifuged at 1,000 rpm for 5 min at 4 °C. Cell pellets were resuspended in ice-cold RPMI-1640 medium containing Matrigel (100 μL per injection) and adjusted to 5 × 10^6^ cells per mouse. Cages were labeled according to experimental groups, the mice were randomly divided into five groups (Control Groups:NC-HGC-27 and OE-VSIR; Drug treatment groups: OE-VSIR + HER2 inhibitor, OE-VSIR + AXL inhibitor, OE-VSIR + HER2 inhibitor + AXL inhibitor), with five mice in each group (*n* = 5 per group). All injections were performed in a UV-sterilized biosafety cabinet. Cells were implanted subcutaneously into the forelimb, and the injection site was gently compressed for 2 min to minimize leakage. Oral drug administration began 24 h post-inoculation and continued daily for 14 days: HER2 (Tucatinib (Irbinitinib) inhibitor (schedule: 200 mg/kg/day; vendors:MedChemExpress (MCE);catalog numbers:HY-16069; Route: PO) in 10% DMSO / 90% (20% SBE-β-CD in saline). The recommended dose of the reagent package was followed in combination with the dose used in the reference; AXL(AXL-IN-13) inhibitor (schedule: 100 mg/kg/day; vendors:Shanghai yuanye Bio-Technology Co., Ltd;catalog numbers:V38509;Route: PO) in vehicle (7.14% Cremophor EL, 2.85% ethanol, 90% saline, with trace 0.1 M HCl). The recommended dose of the reagent package was followed in combination with the dose used in the reference.^34^ Tumor formation was visually confirmed on day 2, and tumor dimensions were measured weekly from day 10 using digital calipers. Tumor volume was calculated as: Volume = ½ × (width^2^ × length). Mice were euthanized on day 30 or earlier if tumor diameter exceeded 1.5 cm. Excised tumors were fixed in 4% paraformaldehyde for immunohistochemical analysis.

#### Cell lines

HEK293T, HGC-27, and MKN-45 cell lines were sourced from the American Type Culture Collection (ATCC) and authenticated by the provider, with certification documentation available. Bone marrow-derived macrophages (BMDMs) were generated by isolating bone marrow cells from murine femurs and tibias, followed by differentiation in RPMI 1640 medium (Gibco) supplemented with 10% fetal bovine serum (FBS), L-glutamine, and 30% L929 cell-conditioned supernatant for 7 days at 37 °C under 5% CO_2_. All cells maintained in DMEM containing 10% FBS, 2 mM L-glutamine, 100 U/mL penicillin, and 100 μg/mL streptomycin under standard incubation conditions (37 °C, 5% CO_2_).

All cell lines used in this study were regularly tested for mycoplasma contamination using the MycoAlert Mycoplasma Detection Kit (Lonza, USA) and were confirmed to be mycoplasma-free. STR profiling was performed for cell line authentication (Data S4, S5, S6, and S7).

### Method details

#### Lentivirus, reagents and transfection experiments

VSIR overexpression and knockdown lentiviruses were constructed, sequenced and packaged by Gene Pharma Biotechnology Co., Ltd. (Shanghai, China). Stable cell lines with overexpression or knockdown of VSIR were constructed in accordance with the Lentivirus Stable Cell Line Construction Manual. To establish VSIR-overexpressing HGC-27 cell lines, cells were infected with recombinant lentiviral vectors carrying the full-length human VSIR coding sequence or an empty vector control. Subsequently, the stably transfected cells were screened using puromycin (Sigma - Aldrich, USA), and the stably transfected cell lines were selected for further experimental analysis.

#### Wound healing assay

Human gastric cancer MKN-45 cells were divided into KDNC-VSIR, KD-VSIR and KD-VSIR+OE-AXL groups, seeded in 6-well plates and cultured to full confluence. Uniform linear scratches were generated vertically across monolayers using sterile 200 μL pipette tips; detached cells were washed off with pre-warmed PBS, followed by serum-free medium replacement. Identical fixed visual fields were photographed under inverted microscope at 0 h and 72 h post-scratching. Scratch closure area was quantified via ImageJ to evaluate cell migratory capacity. Three biological replicates were performed for statistical analysis.

#### Transwell assay

Human gastric cancer MKN-45 cells were classified into KDNC-VSIR, KD-VSIR and KD-VSIR+OE-AXL groups. For migration assay, equal cell suspensions in serum-free medium were seeded into uncoated Transwell upper chambers, while lower chambers contained 10% FBS complete medium as chemoattractant. For invasion detection, chambers were pre-coated with Matrigel matrix. After 24 h incubation, non-migratory/non-invasive upper-surface cells were wiped away. Fixed and crystal violet-stained transmembrane cells were imaged and counted via ImageJ. Three independent biological repeats were conducted for statistical comparison of migratory and invasive phenotypes.

#### Real-time RT-PCR

Total RNA was isolated using RNAiso Plus (Takara). One microgram of DNase-free RNA was reverse-transcribed into cDNA using HiScript II Q RT SuperMix with gDNA wiper (Vazyme). Quantitative real-time PCR (qRT-PCR) was performed on a StepOne Plus system (Applied Biosystems) using ChamQ Universal SYBR qPCR Master Mix (Vazyme). Expression levels of target genes were normalized to GAPDH as the endogenous reference gene. Primer sequences are provided in Table S3.

#### Co-immunoprecipitation assay

HEK293T cells transfected with VSIR-HA were harvested 48 h post-transfection at subconfluence from 10-cm tissue culture dishes. Cells were lysed on ice or by gentle rotation at 4 °C for 30–60 min in 1 mL RIPA buffer (50 mM Tris-HCl, pH 7.4; 150 mM NaCl; 1% Triton X-100; 0.5% sodium deoxycholate; 1 mM EDTA) supplemented freshly with protease inhibitor cocktail (Roche). Lysates were clarified by centrifugation at 18,000 × g for 10 min at 4 °C. The supernatants were incubated overnight with 60 μL anti-HA affinity beads. Following incubation, beads were washed sequentially three times with ice-cold wash buffer (50 mM Tris-Cl, pH 7.4; 300 mM NaCl; 0.1% Triton X-100; 5 mM EDTA; 1× freshly added protease inhibitors) and once with cold PBS. Beads were then immobilized using a magnetic rack, and the supernatant was carefully removed, and bound proteins were eluted by boiling in SDS-PAGE loading buffer, resolved by electrophoresis, and analyzed by Western blotting.

#### IB analysis and antibodies

IB analysis was performed with specific antibodies (described below) and secondary anti-mouse or anti-rabbit antibodies conjugated to horseradish peroxidase (HRP) (described below). Visualization was achieved using chemiluminescence. The following antibodies were used for immunoaffinity purification (IP), IB and immunofluorescence (IF): mouse monoclonal antibody VSIR (Cell Signaling, 54979;1:1,000 for IB, 1:200 for IF); rabbit monoclonal antibody to AXL(Cell Signaling, 8661; 1:1,000 for IB, 1:200 for IF); rabbit monoclonal to STAT3 (Cell Signaling, 12640; 1:1,000 for IB); rabbit monoclonal to p-STAT3 (Cell Signaling, 9145; 1:1,000 for IB) ; mouse monoclonal to p65 (Cell Signaling, 6956; 1:1,000 for IB); rabbit monoclonal to p-p65 (Cell Signaling, 3033; 1:1,000 for IB); rabbit monoclonal to JAK1 (Cell Signaling, 3344; 1:1,000 for IB);rabbit monoclonal to p-JAK1 (Cell Signaling, 74129; 1:1,000 for IB); rabbit monoclonal to GAPDH (Cell Signaling, 5174; 1:1,000 for IB); rabbit monoclonal antibody to HA(Cell Signaling, 3724; 1:1,000 for IB); rabbit monoclonal to HER2(;Fuzhou Maixin Biotechnology Co, 1:2,000 for IB, 1:200 for IP); rabbit monoclonal to Ki-67 (HUABIO, HA721115;1:500 for IHC).

#### Immunofluorescence staining

Formalin-fixed, paraffin-embedded (FFPE) tissue sections were processed for multiplex immunofluorescence using a commercial kit (Ningbo Yangming Medical Laboratory Co., Ltd., China) following the manufacturer’s instructions. After deparaffinization and heat-induced antigen retrieval, sections were blocked to reduce non-specific binding and incubated overnight at 4 °C with primary antibodies. The next day, slides were washed and sequentially treated with HRP-conjugated secondary antibodies and tyramide signal amplification (TSA) reagents labeled with spectrally distinct fluorophores. Nuclei were counterstained with DAPI, and slides were mounted with anti-fade medium and sealed for preservation. For cell-based immunofluorescence, MKN-45 cells seeded on glass coverslips were transfected with plasmids or exposed to indicated stimuli as specified in the figure legends. Cells were then fixed with 4% paraformaldehyde (PFA) for 15 min, permeabilized with 0.1% Triton X-100 in PBS for 5 min, and blocked in PBS containing 5% goat serum and 0.1% Triton X-100 for 30 min at room temperature. Primary antibodies were applied for 2 h, followed by incubation with fluorophore-conjugated secondary antibodies. Nuclei were counterstained with DAPI, and coverslips were mounted onto glass slides. Images were acquired using a LEICA TCA SP8 confocal microscope.

#### ChIP-qPCR

ChIP-qPCR analysis for STAT3 was performed according to standard procedures.^35^ HGC-27 cells were plated in 150 mm tissue culture dishes (1.5×10^7^ cells/dish) and allowed to grow to 80–90% confluency. Cells were then fixed with 1% formaldehyde for 10 min, followed by quenching with 0.125 M glycine for 5 min. Chromatin isolated (∼100 μg) was subjected to sonication to achieve an average fragment size of 300-400 bp. For immunoprecipitation, 10 μg of anti-STAT3 antibody was incubated with 100 μL of Protein G Dynabeads (Thermo Fisher, #10003D) per sample, strictly adhering to the manufacturer’s protocol.

#### Plasmids and transfection

Expression plasmids encoding human VSIR (h-VSIR) and STAT3 (h-STAT3) were generated by amplifying the respective cDNAs via standard PCR and cloning them into the pcDNA3.1(-) vector. To construct the AXL reporter plasmid, a putative enhancer region was inserted upstream of the minimal promoter in the pGL3-Promoter vector (Promega). All constructs were verified by Sanger DNA sequencing. For transient transfections, HEK293T cells were transfected with plasmids using linear polyethylenimine (PEI). HGC-27 cells were transfected with plasmid DNA or si-RNA using jetPRIME (polyplus) according to the manufacturer’s instructions. si-RNA target sequences are shown in Table S3.

#### Dual-luciferase reporter assay

To assess the transcriptional enhancer activity of a candidate genomic region from the AXL locus, a DNA fragment containing the wild-type (WT) sequence (chr19:41225274-41227133) and a mutant (mut) variant with the sequence “CAT CTG GAA AG”(a predicted STAT3 binding site identified by JASPAR) mutated to “ACC ACA ACC CA” were synthesized by Tsingke Biotechnology (Beijing, China) and subsequently cloned into the multiple cloning site (MCS) upstream of the SV40 minimal promoter in the pGL3-Promoter vector (Promega, Madison, WI). HEK293T cells were seeded in 24-well plates at a density of 5 × 10^4^ cells per well and cultured overnight to reach 70-80% confluence. Cells were co-transfected with 500 ng of the AXL enhancer reporter plasmid, along with either an empty vector or expression plasmids STAT3, together with 20 ng of the Renilla luciferase control plasmid (pRL-TK; Promega). Transfections were performed using linear polyethylenimine (PEI, 25 kDa) at a DNA:PEI ratio of 1:3 (w/w) in Opti-MEM medium. After 6 h, the transfection mixture was replaced with fresh complete growth medium. Luciferase activities were measured 24 h post-transfection using the Dual-Luciferase® Reporter Assay System (Promega) according to the manufacturer’s instructions. Briefly, cells were lysed in 1× Passive Lysis Buffer, and Firefly and Renilla luciferase activities were sequentially quantified on a luminometer. Enhancer activity was calculated as the ratio of Firefly to Renilla luciferase signal and normalized to the activity of the empty pGL3-Promoter vector control.

#### RNA sequencing

In this study, a heatmap was employed to visualize and analyze the gene expression/microbial species abundance matrix acquired through high - throughput sequencing. The raw count matrix underwent preprocessing, which involved filtering out low - abundance features, and was standardized using the Z - score method to eliminate dimensionality and emphasize relative variation among samples. Subsequently, based on the processed data, the Euclidean distance was utilized to compute the similarity between samples and features, and the complete linkage method was applied for hierarchical clustering to systematically uncover the intrinsic clustering structure of the data. The visualization was accomplished using the pheatmap package (version: packageVersion('pheatmap') [1] '1.0.12') in the R language (version: R (4.5.0)), and the standardized numerical distribution was visually represented via a designed color gradient (e.g., blue - white - red gradient). Simultaneously, sample grouping annotation bars, row - line clustering dendrograms, and feature significance markers (∗ indicating *P* < 0.05) were incorporated into the heatmap to clearly display the difference characteristics and association patterns between groups.

#### Heatmap analysis and visualization

Heatmaps were generated to visualize gene expression profiles and microbial species abundance matrices derived from high-throughput sequencing. Raw count matrices underwent preprocessing, which included filtering out low-abundance features followed by Z-score normalization to eliminate dimensional effects and highlight relative variations across samples. Subsequently, hierarchical clustering was performed on the processed data using Euclidean distance to calculate similarity between samples and features, coupled with the complete linkage method to systematically reveal intrinsic clustering structures. All visualizations were implemented using the pheatmap package (version 1.0.12) in R (version 4.5.0). A diverging color gradient (ranging from blue to white to red) was applied to intuitively map the distribution of normalized values. To clearly present inter-group differential features and association patterns, the heatmaps were annotated with sample grouping bars, row and column dendrograms, and significance markers (*p* < 0.05).

### Quantification and statistical analysis

The positive rate and area ratio of immunohistochemistry and immunofluorescence multi-label experiments were analyzed by HALO image analysis software (version: Halo 3.4.2986, company: Lndica Labs). All data are presented as mean ± standard deviation (s.d.). Statistical significance was assessed using a two-tailed, unpaired Student’s t-test unless otherwise specified in the corresponding figure legends. *p* ≤ 0.05 was adopted as the threshold for statistical significance. The sample size (n), denoting the number of mice per experimental group, is explicitly stated in each figure legend. For experiments involving comparisons across more than two groups, either Dunnett’s post hoc test following one-way ANOVA or two-way ANOVA was applied, as detailed in the relevant figure legends. In these analyses, statistical differences with a *p* ≤ 0.05 was considered significant.

## Supplemental information

Document S1. Tables S1–S3

Data S1. Animal ethics approval documentation supporting the animal experiments described in STAR Methods

Data S2. Patient ethics approval documentation dated September 14, 2025, supporting the human tissue study described in STAR Methods

Data S3. Patient ethics approval documentation (2026–0273-01) supporting the human tissue study described in STAR Methods

Data S4. STR identification report for 293T cells, related to STAR Methods

Data S5. STR identification report for HGC-27 cells, related to STAR Methods

Data S6. STR identification report for MKN-45 cells, related to STAR Methods

Data S7. STR identification report for THP-1 cells, related to STAR Methods
