# Supplementary material for: Mechanistic insights into VSIR-mediated AXL regulation and gastric cancer proliferation
Source: iScience. 2026 Sep 18;29(10):117580. doi: 10.1016/j.isci.2026.117580 (PMC13626921; doi:10.1016/j.isci.2026.117580)
Supplement: Supplementary file 1 [file mmc1.pdf]

iScience, Volume *29*

Supplemental information

Mechanistic insights into VSIR-mediated AXL regulation and gastric cancer proliferation

Jinshan Liu, Guoquan Huang, Yi Li, Yong Tan, Bo Qin, Changjiang Hao, Jilv Peng, Hongyang Zhu, Bitao Zhang, Yong Cheng, and Kun Qian

SUPPLEMENTAL INFORMATION

Table S1: The basic clinical information of 3 gastric cancer patients in Figure 6A and Figure 6B immune multi-label experiments was collected.

PATIENT ID	SEX	AGE (YEARS)	ETHNICITY	TNM STAGE
1	Man	66	Han nationality	T4aN3M0
2	Man	59	Han nationality	T4aN1M0
3	Man	62	Han nationality	T4aN3M0

Table S2: The basic clinical information of 6 gastric cancer patients in Figure 6c-Figure 6F immune multi-label experiment, in which the normal gastric mucosa tissues in Figure 6E are paired normal samples of cancer tissues in ID4-6 patients.

PATIENT ID	SEX	AGE (YEARS)	ETHNICITY	HER2 NEGATIVE/POSITIVE	HER2-TARGETED THERAPIES
1	Man	55	Han nationality	Positive	Videcital
2	Man	57	Han nationality	Positive	Videcital
3	Man	67	Han nationality	Positive	Videcital
4	Man	78	Han nationality	Positive	NO
5	Man	56	Han nationality	Positive	NO
6	Female	57	Han nationality	Positive	NO
7	Man	73	Han nationality	Negative	NO
8	Female	72	Han nationality	Negative	NO
9	Man	66	Han nationality	Negative	NO

Table S3: This study involved qRT-PCR primers for genes, si-RNA, gene overexpression knockdown or overexpression plasmid/lentivirus sequences, details are as follows:

REAGENT OR RESOURCE	SOURCE	IDENTIFIER
Oligonucleotides		N/A
siRNA targeting sequence:		N/A

si-hVSIR#1: CCUUGAAUGUUGCUGUCCAUCTT<br>si-hVSIR#2: UCUUUGCCUGUCUGCACCUGCTT	Wuhan Hecegene Technology Co.,Ltd (Wuhan, China)	N/A
si-hSTAT3#1: CUUCAGACCCGUCAACAAATT<br>si-hSTAT3#2: GGAGCUGUUUAGAAACUUATT<br>si-hSTAT3#3: UCACAUGCCACUUUGGUGUTT	GenePharma (Shanghai, China)	N/A
si-hTLR4: GAGCCGCUGGUGUAUCUUUTT	Wuhan Hecegene Technology Co.,Ltd (Wuhan, China)	N/A
si-mSTAT3: GGGUCUGGCUAGACAAUAUTT	GenePharma (Shanghai, China)	N/A
Primer: VSIR<br>Forward: GAGATCAGGCACCACCACTC<br>Reverse: TGCCTGCCTTTGCTTGTAGA	This paper	N/A
Primer: AXL<br>Forward: CCGTGGACCTACTCTGGCT<br>Reverse: CCTTGGCGTTATGGGCTTC	This paper	N/A
Primer: AXL(Pre-mRNA)<br>Forward: CCTCACCTCGCAAGCATCC<br>Reverse: TGCCACGCGGATGTGATAAG	This paper	N/A
Primer:STAT3<br>Forward: GGCATTCGGGAAGTATTGTCG<br>Reverse: GGTAGGCGCCTCAGTCGTATC	This paper	N/A
Primer:IL6<br>Forward: ACTCACCTCTTCAGAACGAATTG<br>Reverse: CCATCTTTGGAAGGTTCAGGTTG	This paper	N/A
Primer: GAPDH<br>Forward: GGAGTCCACTGGCGTCTTCA<br>Reverse: GTCATGAGTCCTTCCACGATACC	This paper	N/A

Overexpresses the lentiviral sequence

ID	SEQ
VSIR(83930-1)-p1	AGGTCGACTCTAGAGGATCCCGCCACCATGGGCGTCCCCACGGCCCTG
VSIR(83930-1)-p2	TCCTTGTAGTCCATACCGATGACCTCAAAGTTTGGAGAGTC

VSIR knockdown of the lentiviral sequence

CONTINUED

NO.	5’	STEM	Loop	STEM	3’

VSIR-RNAi(112434-1)-a	Ccgg	CCCTGACTCTCCAAACTTTGA	CTCGAG	TCAAAGTTTGGAGAGTCAGGG	TTTTTg
VSIR-RNAi(112434-1)-b	aattcaaaaa	CCCTGACTCTCCAAACTTTGA	CTCGAG	TCAAAGTTTGGAGAGTCAGGG	
VSIR-RNAi(112435-1)-a	Ccgg	CCGTATTCCCTGTATGTCTGT	CTCGAG	ACAGACATACAGGGAATACGG	TTTTTg
VSIR-RNAi(112435-1)-b	aattcaaaaa	CCGTATTCCCTGTATGTCTGT	CTCGAG	ACAGACATACAGGGAATACGG	
VSIR-RNAi(112436-1)-a	Ccgg	CGGATGGACAGCAACATTCAA	CTCGAG	TTGAATGTTGCTGTCCATCCG	TTTTTg
VSIR-RNAi(112436-1)-b	aattcaaaaa	CGGATGGACAGCAACATTCAA	CTCGAG	TTGAATGTTGCTGTCCATCCG	
