# Supplementary material for: Mechanistic insights into VSIR-mediated AXL regulation and gastric cancer proliferation
Source: iScience. 2026 Sep 18;29(10):117580. doi: 10.1016/j.isci.2026.117580 (PMC13626921; doi:10.1016/j.isci.2026.117580)
Supplement: Supplementary file 2 [file mmc2.pdf]

# 重庆医科大学附属第一医院伦理委员会

## 审批意见书

**申请人:**钱昆

**申请审批项目名称:**基于单细胞测序技术研究 VSIR 基因在胃癌形成过程中促使胃癌细胞产生免疫逃逸的机制研究

**审查日期:**2023 年 04 月 26 日

**批准日期:**2023 年 04 月 26 日

**审查批号:**2023 年科研伦理（K2023-100）

---

经伦理委员会审查，本项目所提交的研究方案及知情同意书等资料符合医学伦理原则和赫尔辛基宣言的各项要求，研究设计具有科学根据，没有给受试者带来不必要的危险，对受试者的安全和隐私给予了最大限度的保护，批准该项目按预定方案实施。

重庆医科大学附属第一医院伦理委员会

2023 年 04 月 26 日

重庆医科大学附属第一医院伦理委员会

**研究方案说明**

**研究方案可围绕如下要点，进行陈述。**

- **研究的背景：**需要注意在国际合作中，此研究项目对当地的需求和贡献的考虑；鼓励在后面附参考文献。
- 胃癌在全世界范围内高发。据数据统计，胃癌的发病率居恶性肿瘤第五位，死亡率居第三位，其五年生存率仅为 5%-15%。据 2020 年数据统计，我国胃癌年发病 48 万，占中国全部癌症发病率的第三位，约占世界胃癌患者的 44%；其 5 年生存率仅为 35.1%，占中国全部癌症死亡率的第 2 位，死亡病例数约占全世界的 50%。近年来，随着医疗水平的不断发展，胃癌的诊治水平也有所提高，但胃癌患者的早期诊断和治疗水平仍然未得到明显改善。我国胃癌患者行手术及相关治疗后，其 5 年生存率仍然未超过 40%。胃癌的形成、侵袭和转移是一个涉及到多个基因参与、且共同作用的复杂过程。包括癌基因突变或异常过表达、抑癌基因失活、肿瘤细胞产生免疫逃逸等等。多种基因的参与促进了胃癌细胞的恶性表型，增加了胃癌细胞的异质性、诱导肿瘤血管生成、逃避宿主的免疫监视，促使肿瘤细胞产生免疫逃逸，最终导致胃癌细胞无限增殖并形成胃癌或是转移灶。目前全世界针对胃癌的形成机制的研究尚欠。因此，需要我们深入探讨胃癌形成的分子机制，同时寻找到胃癌诊断和治疗的新靶点。
- （1）免疫系统与胃癌的形成

- 正常的免疫系统可以清除人体内有缺陷、死亡或是凋亡的细胞。可在胃癌患者中，胃癌细胞是如何逃脱机体的免疫监视，从而得以存活并最终形成胃癌。目前研究表明：肿瘤在形成过程中产生的免疫应答是一种极其复杂的动态现象，它在肿瘤的形成和消退过程中起到了至关重要的作用。Dunn 等人提出了“3E 免疫编辑”学说,即:清除、平衡和逃逸(Eliminate,Equilibrium and Escape)。一些肿瘤细胞通过免疫编辑，使肿瘤细胞和免疫系统达到平衡状态,但是一旦机体在免疫系统能力出现低下时，处于隐匿状态的肿瘤细胞就会战胜机体的免疫系统。当胃癌细胞在与机体发生免疫系统斗争的过程中，癌细胞在肿瘤微环境中定植、发展并增殖。抑制、耐受和逃避被识别是胃癌发生逃避免疫监视的方式。近年来，免疫治疗取得了快速发展，如靶向促进肿瘤形成和转移的免疫细胞，阻断辅助逃避免疫监视的细胞表面蛋白,以及抑制肿瘤微环境生成细胞因子和趋化因子。PD-1/PDL1 免疫疗法是当前备受瞩目的一类抗癌免疫疗法,旨在利用人体自身的免疫系统抵御肿瘤,通过阻断 PD-1/PD-L1 信号通路使肿瘤细胞死亡,适用于多种类型肿瘤。但是目前针对胃癌的免疫治疗尚未取得突破性进展，需要我们进一步寻找胃癌的免疫治疗和检测的新靶点。单细胞测序技术对于肿瘤免疫的机制研究提供了更精确的新证据
- 在过去的几十年里，免疫治疗已经被认为是治疗多种人类癌症最有效的办法[1]。在免疫治疗的策略中，免疫检查点的封锁正

在成为癌症免疫治疗的最前沿方法，也是最有效的方法[2]。已经进行了大量研究来阐明代表性免疫检查点的机制和治疗潜力，如 CTLA-4 和 PD-1。此外，具有免疫检查点活性的新靶点的发现为肿瘤免疫调节网络的系统研究提供了新的机会，也为开发有前景的治疗药物提供了潜在的突破。VSIR 基因是 T 细胞活化的 V 结构域免疫球蛋白抑制因子，又称 VISTA[3]。VISTA 是一种 I 型跨膜蛋白，由一个 n 端免疫球蛋白(Ig) v 结构域、大约 30 个氨基酸组成的柄(aa)、一个跨膜结构域和 95-aa 的细胞质尾部组成[4]。VSIR 蛋白表达水平最高的是髓系细胞，尤其是小胶质细胞和中性粒细胞，其次是单核细胞、巨噬细胞和树突状细胞[5]。VISTA 被认为是一种 V-set 受体，在几种人类癌症中，如前列腺癌、非小细胞肺癌(NSCLC)和结直肠癌中，可以抑制 T 细胞相关的免疫逃逸和生存反应[6-8]。VISTA 也在激活抗癌免疫反应中扮演着类似检查点的刺激角色。由于免疫治疗为很多癌症的治疗提供了新的方向，目前关于胃癌的免疫治疗药物极度缺乏，因此寻找胃癌新的免疫检查点，为胃癌开发新的免疫治疗药物，变得非常重要。目前我省关于胃癌的免疫治疗的探索极少，针对胃癌形成的免疫方面的研究更是少之又少。目前关于胃癌形成过程中，胃癌细胞产生免疫逃逸的机制尚不明确。本课题基于单细胞测序技术，将临床胃癌患者的原发灶和癌旁正常组织解离成单细胞，然后通过 10×测序技术平台，分析胃癌原发灶和癌旁正常组织中关于免疫相关标志基因，

寻找胃癌形成过程中新的免疫检查点，为胃癌的诊疗提供新的理论依据。在分析单细胞测序数据时发现 VSIR 基因在胃癌形成过程中，扮演了非常重要的角色。但是关于 VSIR 基因在胃癌形成过程如何帮助胃癌细胞实现免疫逃逸的机制尚不明确，需要进一步研究。因此我们将研究 VSIR 基因在肿瘤微环境中的作用以及 VSIR 基因是如何促使胃癌细胞产生免疫逃逸进而导致胃癌形成的机制作为本项目的主要研究对象。

- 参考文献


- [1]:Mellman I, Coukos G, Dranoff G. Cancer immunotherapy comes of age.
- Nature. 2011;480(7378):480 - 9.
- [2]:Tang J, et al. Trial watch: the clinical trial landscape for PD1/PDL1 immune checkpoint inhibitors. Nat Rev Drug Discov. 2018;17(12):854 - 5.
- [3]:Wang L, et al. VISTA, a novel mouse Ig superfamily ligand that negatively regulates T cell responses. J Exp Med. 2011;208(3):577 - 92.
- [4]:Flies DB, et al. Cutting edge: a monoclonal antibody specific for the
- programmed death-1 homolog prevents graft-versus-host disease in mouse

- models. J Immunol. 2011;187(4):1537–41.
- [5]:Borggrewe M, et al. VISTA expression by microglia decreases during
- inflammation and is differentially regulated in CNS diseases. Glia. 2018;
- 66(12):2645–58.
- [6]:Villarroel-Espindola F, et al. Spatially resolved and quantitative analysis of VISTA/PD-1H as a novel immunotherapy target in human non-small cell lung cancer. Clin Cancer Res. 2018;24(7):1562–73.
- [7]:Gao J, et al. VISTA is an inhibitory immune checkpoint that is increased after ipilimumab therapy in patients with prostate cancer. Nat Med. 2017;23(5):551–5.
- [8]:Xie S, et al. Expression of the inhibitory B7 family molecule VISTA in human colorectal carcinoma tumors. Cancer Immunol Immunother. 2018;67(11):1685–94.
- 目前本人有独立的研究团队，本人有能力指导所有基础实验。本研究旨在于寻找胃癌新的免疫检查点和胃癌早期诊断和治疗的新靶点。

  **研究的目的和预期成果：**

  预期发表 2-3 篇高质量的 SCI 论文。申请专利一项。
- **研究的设计和方法：**本研究为临床对照实验：采用需行手术治

疗的胃癌患者的临床样本约 100 例，在胃癌患者切下的手术标本上取癌组织和癌旁正常组织，进行单细胞测序、免疫组化、qRT-PCR、蛋白质 4D 技术、WB 等实验，深层次研究胃癌的发病机制，为胃癌患者谋福利。

- **研究实施的过程：**鼓励绘出流程图。需要持续的时间、具体的干预的方法，等。
- A：单细胞数据的基础分析技术路线图

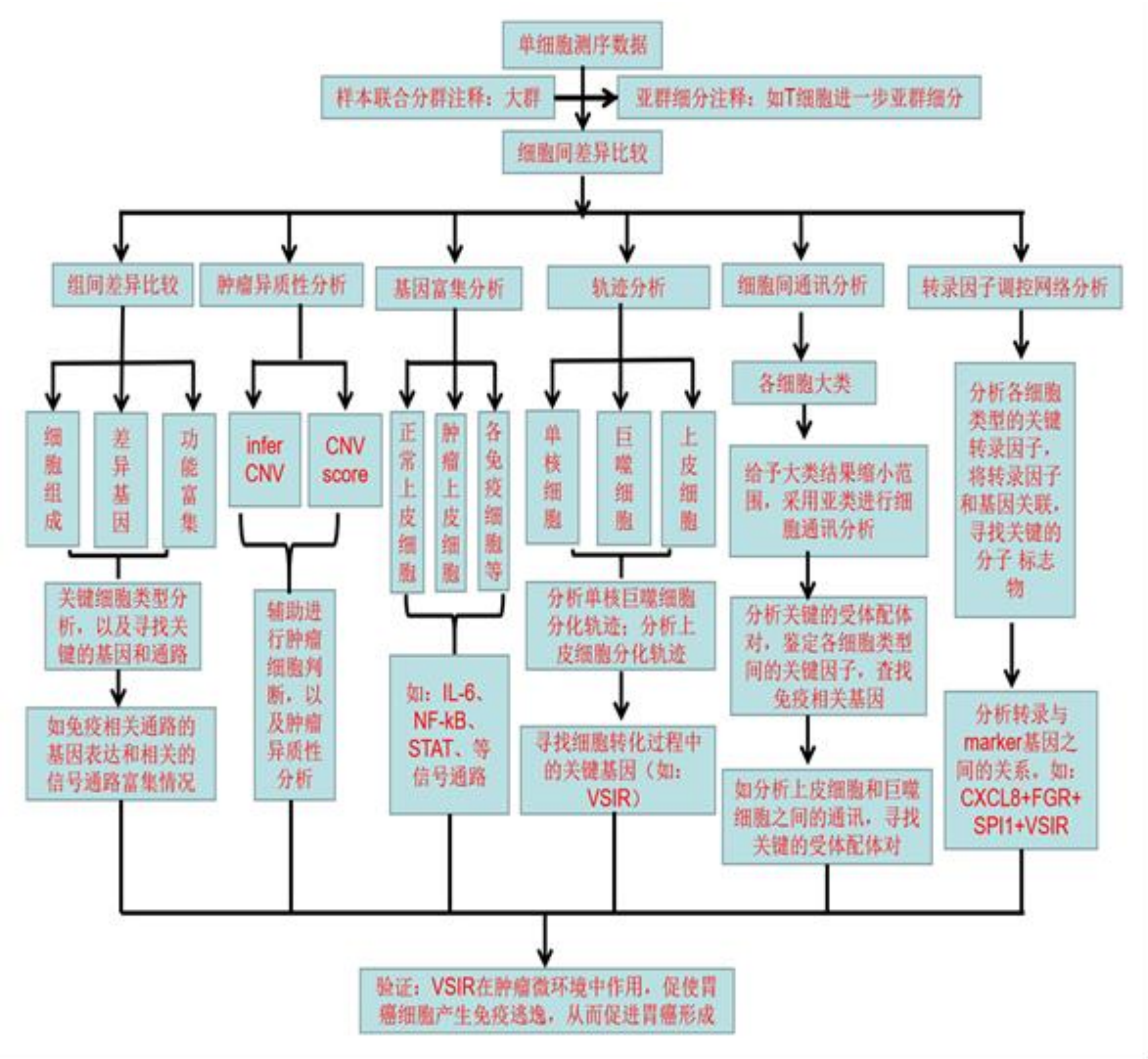


- 
- B：基础实验验证 VSIR 基因在胃癌形成过程中的作用，技术路线图如下：

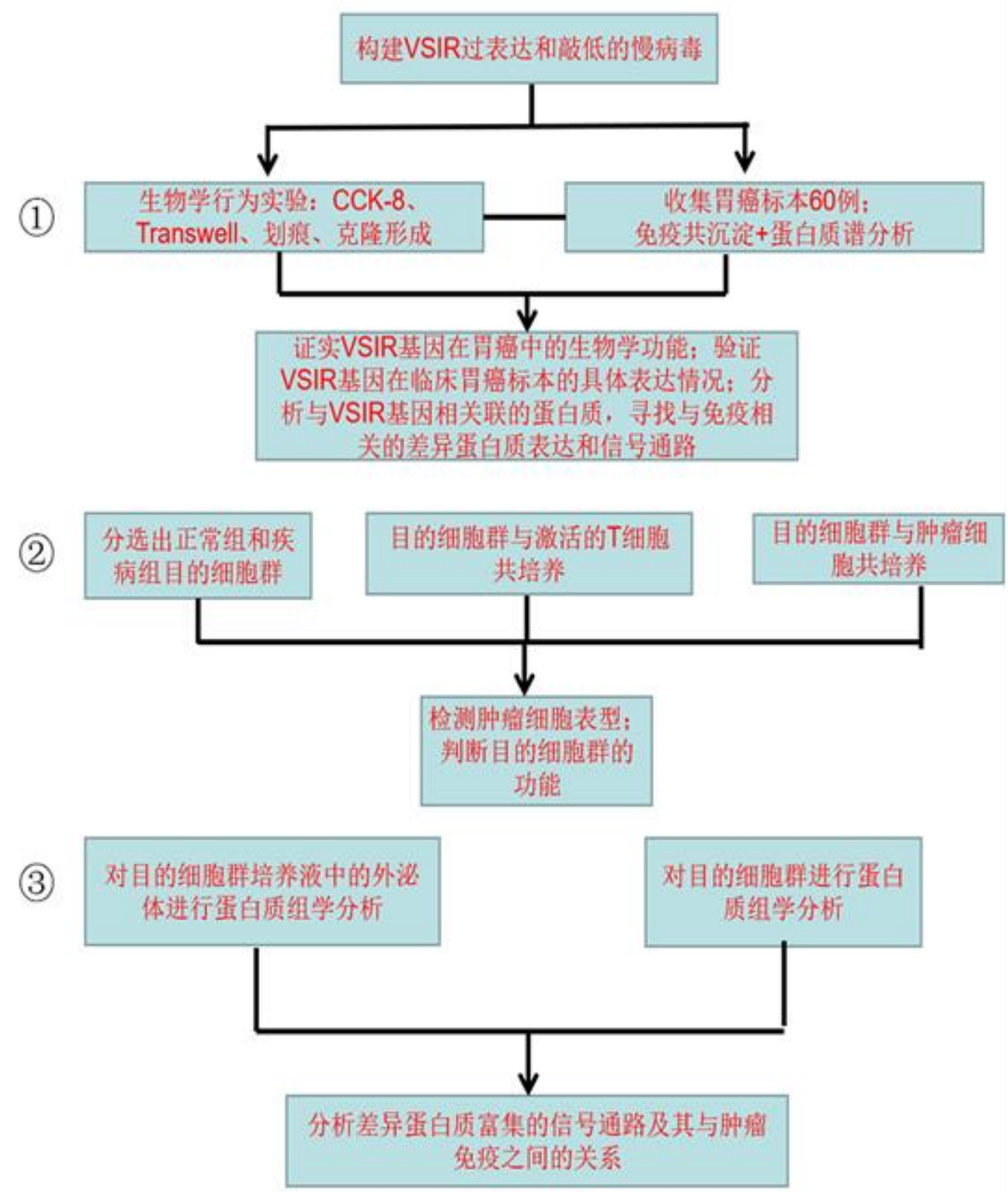


- 
- C:在老鼠体内证实VSIR基因导致胃癌形成，技术路线图如下：

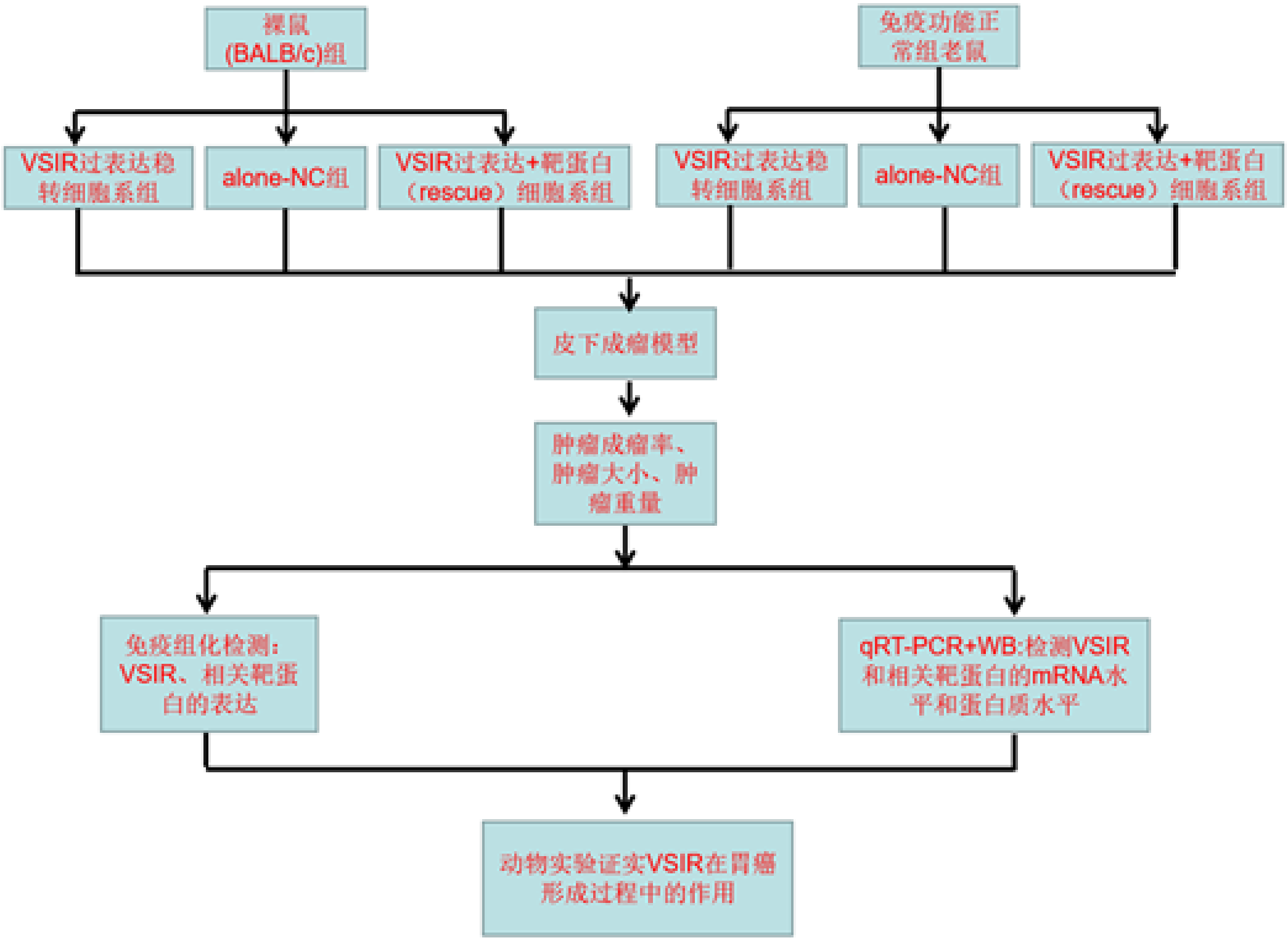


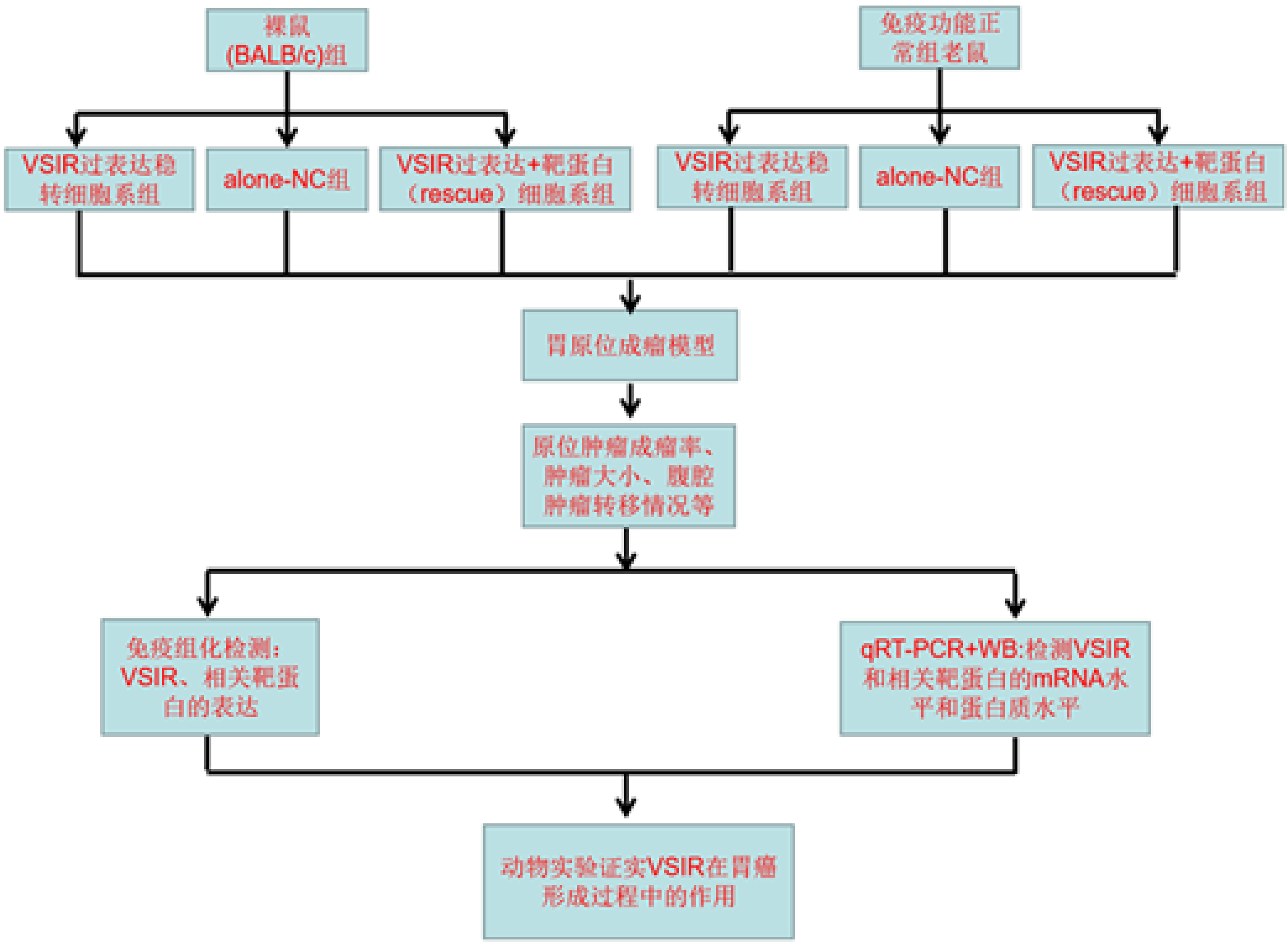


- 
- **研究的风险/收益：**经过本项目的研究，寻找到关于胃癌新的免疫检查点，然后与相关药企合作，实现对胃癌的免疫靶向治疗，为胃癌患者带来巨大福音，造福社会、造福人类，同时也可以

实现巨大的经济价值。研究采用的是患者手术切下的标本，对患者的诊断和治疗不会造成任何影响，也不会增加患者的经济负担。

- **受试者的招募和保护措施：**本研究使用患者行胃癌手术时切下的手术标本进行研究，无需进行受试者招募。
- **知情同意过程：**在患者与主治医师共同确定手术方案后，由研究员单独与患者或患者的代理人进行沟通，并签署知情同意书。
- **对受试者是否有补偿，哪些能免费，哪些会得到补偿，和哪些需要付费的说明。**本研究不会影响患者的诊断和治疗，不会增加患者的治疗负担，因此不对患者进行补偿。
- 数据的获得和质控：研究数据主要为单细胞测序数据，由通过测序机构完成并由专业的研究人员进行指控。
- **有关受试者的信息和保密**：本研究不会泄露患者的个人数据，医疗档案及病理标本仅用于本次研究
- 成果分享：测序结果或研究结果可能上传公共数据库，可能通过发表文章进行分享。

说明：

此处提供的要点，可作为研究者自己撰写研究方案的参考。要以完整方案的文档呈报，而非以对此要点文档的填空或者问答的方式递交伦理委员会。

方案内容不限于这些内容，需补充必要的相关内容，如有关心理测量的，需要提交使用的量表；做问卷调查的，需要提交调研问卷本

身；或者有些内容可能不需涉及。

鼓励用自己的话语通俗易懂地描述出来。需注意不要与知情同意书中的内容重复。

# 知情同意书

**研究背景介绍（简写）：**胃癌是威胁人类健康的主要消化道肿瘤之一。胃癌的形成、侵袭和转移与肿瘤细胞免疫逃逸关联。对胃癌原发灶和癌旁正常组织进行单细胞测序。单细胞测序结果表明：VSIR 基因在胃癌组织中明显升高，在巨噬细胞和单核细胞中的表达量异常升高，提示VSIR 基因在胃癌的形成过程中发挥了重要作用。本课题拟在单细胞测序的基础上，分析 VSIR 在胃癌形成中如何使胃癌细胞产生免疫逃逸的作用机制。然后通过体内外实验及蛋白质 4D 技术研究VSIR在胃癌形成过程中的机制。

尊敬的患者（受试者）：

您好！

由于您是胃癌患者，需要进行手术治疗，您将被邀请参加一项由重庆医科大学附属第一医院胃肠外科钱昆教授主持的研究。这是为探索VSIR 在胃癌形成过程中的机制而进行一项研究，它将历时 3 年时间。此项目由重庆市科技厅重大项目资助进行。

本知情同意书提供给您一些信息以帮助您决定是否参加此项临床研究。您参加本项研究是自愿的。本次研究已通过本研究机构伦理审查委员会审查。如果你同意加入此项研究，请看下列说明：

请您仔细阅读，如有任何疑问请向负责该项研究的研究者提出。

**研究目的：（背景意义—简写）**

胃癌是威胁人类健康的主要消化道肿瘤之一。胃癌的形成、侵袭和转移与肿瘤细胞免疫逃逸关联。对胃癌原发灶和癌旁正常组织进行单细胞测序。单细胞测序结果表明：VSIR 基因在胃癌组织中明显升

高，在巨噬细胞和单核细胞中的表达量异常升高，提示VSIR 基因在胃癌的形成过程中发挥了重要作用。本课题拟在单细胞测序的基础上，分析 VSIR 在胃癌形成中如何使胃癌细胞产生免疫逃逸的作用机制。然后通过体内外实验及蛋白质 4D 技术研究VSIR 在胃癌形成过程中的机制。

**研究过程和方法（简写）：**

如果您同意参与这项研究，我们将对每位受试者进行编号，建立病历档案。在研究过程中我们需要采集一些您的标本，将由专业人员为您取样，我们将从您手术切下的标本上取一小块组织进行研究，您的生物样品仅用于本次研究。

**研究可能的受益：**

经过本项目的研究，寻找到关于胃癌新的免疫检查点，然后与相关药企合作，实现对胃癌的免疫靶向治疗，为胃癌患者带来巨大福音，造福社会、造福人类，同时也可以实现巨大的经济价值。

**研究风险与不适：**在您的手术标本上取一小块组织不会对您后续的诊断和治疗造成任何影响，也不会增加您的治疗费用。

**其他治疗干预方式：**

您除了参加此研究外，无其他的干预或治疗措施。

**隐私问题：**（过程中的隐私保护和结果发表的隐私保护）

如果您决定参加本项研究，您参加试验及在试验中的个人资料均属保密。对于您来说，所有的信息将是保密的。您的病理标本将以研究编号数字而非您的姓名加以标识。可以识别您身份的信息将不会透露给研究小组以外的成员，除非获得您的许可。所有的研究成员和研

究申办方都被要求对您的身份保密。为确保研究按照规定进行，必要时，政府管理部门或伦理审查委员会的成员按规定可以在研究单位查阅您的个人资料。

如果这项研究结果发表时，研究者也需要对保密方面进行承诺。

**费用和补偿：**本研究不会影响您原有的治疗方案及治疗费用。

**自由退出：**

作为受试者，您可随时了解与本研究有关的信息资料和研究进展，自愿决定（继续）参加还是不（继续）参加。参加后，无论是否发生伤害，是否严重，您可以选择在任何时候不需要任何理由通知研究者要求退出研究，您的数据将不纳入研究结果，您的任何医疗待遇与权益不会因此而受到影响。如果继续参加研究，会对您造成严重的伤害，研究者也将会终止研究的进行。

但在参加研究期间，请您提供有关自身病史和当前身体状况的真实情况；告诉研究医生自己在本次研究期间所出现的任何不适；不得服用受限制的药物、食物等；告诉研究医生自己在最近是否曾参与其他研究，或目前正参与其他研究。如果因为您没有遵守研究计划，或者发生了与研究相关的损伤或者有任何其它原因，研究医师可以终止您继续参与本项研究。

**联系方式：**

如果您有与本研究有关的问题，或您在研究过程中发生了任何不适与损伤，或有关于本项研究参加者权益方面的问题，您可以与钱昆，**电话** 18623184180。【项目组的联系人必须为有资质的本院医生】

**试验后利益分享：**

本研究为理论性成果，暂无利益分享。

**知情同意签字：**

我已经阅读了本知情同意书，并且我的医生______________已经将此次临床试验的目的、内容、风险和受益情况向我作了详细的解释说明，对我询问的所有问题也给予了解答，我对此项临床研究已经了解，我自愿参加本项研究。

受试者签名：____________________

日期：　　　年　　月　　日

研究者签名：______________________

日期：　　　年　　月　　日

# 重庆医科大学附属第一医院临床科研伦理委员会

# 伦理审查申请表

<table>
<tr><td colspan="4">项目名称：基于单细胞测序技术研究 VSIR 基因在胃癌形成过程中促使胃癌细胞产生免疫逃逸的机制研究</td></tr>
<tr><td>项目负责人：钱昆</td><td>职称：副教授</td><td colspan="2">研究科室：重庆医科大学附属第一医院胃肠外科</td></tr>
<tr><td colspan="2">项目联系人：钱昆</td><td colspan="2">联　系　电　话　：18623184180</td></tr>
<tr><td colspan="2">合作研究单位：湖北恩施土家族苗族自治州中心医院</td><td colspan="2">联系电话：13469739238</td></tr>
<tr><td>研究者：黄国全</td><td>职称：副教授</td><td>研究者：黎晖</td><td>职称：副教授</td></tr>
<tr><td>研究者：</td><td>职称：</td><td>研究者：</td><td>职称：</td></tr>
<tr><td>研究者：</td><td>职称：</td><td>研究者：</td><td>职称：</td></tr>
<tr><td colspan="4">拟研究时间：2021 年 09 月 10 日 至 2024 年 09 月 10 日</td></tr>
<tr><td colspan="4">研究课题来源： 重庆市科技厅重大项目、湖北省科技厅重大项目</td></tr>
<tr><td colspan="4">递交审查资料：<br>√实验/试验方案　√知情同意书　☐其他资料________________________<br>包括：试验用品安全性资料、生产企业资质证明、试验用品提供者的资质证明</td></tr>
</table>

**研究内容摘要：**

胃癌是威胁人类健康的主要消化道肿瘤之一。胃癌的形成、侵袭和转移与肿瘤细胞免疫逃逸关联。对胃癌原发灶和癌旁正常组织进行单细胞测序。单细胞测序结果表明：VSIR 基因在胃癌组织中明显升高，在巨噬细胞和单核细胞中的表达量异常升高，提示VSIR 基因在胃癌的形成过程中发挥了重要作用。本课题拟在单细胞测序的基础上，分析 VSIR 在胃癌形成中如何使胃癌细胞产生免疫逃逸的作用机制。然后通过体内外实验及蛋白质 4D 技术研究VSIR 在胃癌形成过程中的机制。

**保密要点：**有关受试者的医学记录和研究资料都是保密的。研究结果发表时，与受试者有关的信息资料不会被公开。

**项目负责人签字：钱昆**

**申报科室意见：**

1. 承担项目的研究者是否具备项目所要求的专业知识、经验和时间

☑ 是 □ 否

2. 该研究是否具有创新性、安全性和科学性 ☑ 是 □ 否

3. 该研究是否会给受试者带来额外的经济负担，是否存在过度的或者不合理的检查。 □ 是 ☑ 否

科室负责人签字：钱昆

2021 年 9 月 1 日

填表说明：1、申请日期请填写拟交申请日期。

2、申请书中方格可在文字输入打印后，在选中的项目前用钢笔画√。

3、联系人为：本研究项目的联系人及电话。

4、研究者包括合作研究单位的人员。

HUAWEI Mate X7 | XMAGE
24mm F2.8 1/100s ISO100 | 2026/08/26 11:50 | 重庆市

## 知情同意书

**研究背景介绍（简写）：**胃癌是威胁人类健康的主要消化道肿瘤之一。胃癌的形成、侵袭和转移与肿瘤细胞免疫逃逸关联。对胃癌原发灶和癌旁正常组织进行单细胞测序。单细胞测序结果表明：VSIR 基因在胃癌组织中明显升高，在巨噬细胞和单核细胞中的表达量异常升高，提示VSIR 基因在胃癌的形成过程中发挥了重要作用。本课题拟在单细胞测序的基础上，分析 VSIR 在胃癌形成中如何使胃癌细胞产生免疫逃逸的作用机制。然后通过体内外实验及蛋白质 4D 技术研究VSIR 在胃癌形成过程中的机制。

尊敬的患者（受试者）：

您好！

由于您是胃癌患者，需要进行手术治疗，您将被邀请参加一项由重庆医科大学附属第一医院胃肠外科钱昆教授主持的研究。这是为探索VSIR 在胃癌形成过程中的机制而进行一项研究，它将历时 3 年时间。此项目由重庆市科技厅重大项目资助进行。

本知情同意书提供给您一些信息以帮助您决定是否参加此项临床研究。您参加本项研究是自愿的。本次研究已通过本研究机构伦理审查委员会审查。如果你同意加入此项研究，请看下列说明：

请您仔细阅读，如有任何疑问请向负责该项研究的研究者提出。

**研究目的：（背景意义—简写）**

胃癌是威胁人类健康的主要消化道肿瘤之一。胃癌的形成、侵袭和转移与肿瘤细胞免疫逃逸关联。对胃癌原发灶和癌旁正常组织进行单细胞测序。单细胞测序结果表明：VSIR 基因在胃癌组织中明显升

高，在巨噬细胞和单核细胞中的表达量异常升高，提示VSIR 基因在胃癌的形成过程中发挥了重要作用。本课题拟在单细胞测序的基础上，分析 VSIR 在胃癌形成中如何使胃癌细胞产生免疫逃逸的作用机制。然后通过体内外实验及蛋白质 4D 技术研究VSIR 在胃癌形成过程中的机制。

**研究过程和方法（简写）：**

如果您同意参与这项研究，我们将对每位受试者进行编号，建立病历档案。在研究过程中我们需要采集一些您的标本，将由专业人员为您取样，我们将从您手术切下的标本上取一小块组织进行研究，您的生物样品仅用于本次研究。

**研究可能的受益：**

经过本项目的研究，寻找到关于胃癌新的免疫检查点，然后与相关药企合作，实现对胃癌的免疫靶向治疗，为胃癌患者带来巨大福音，造福社会、造福人类，同时也可以实现巨大的经济价值。

**研究风险与不适：**在您的手术标本上取一小块组织不会对您后续的诊断和治疗造成任何影响，也不会增加您的治疗费用。

**其他治疗干预方式：**

您除了参加此研究外，无其他的干预或治疗措施。

**隐私问题：**（过程中的隐私保护和结果发表的隐私保护）

如果您决定参加本项研究，您参加试验及在试验中的个人资料均属保密。对于您来说，所有的信息将是保密的。您的病理标本将以研究编号数字而非您的姓名加以标识。可以识别您身份的信息将不会透露给研究小组以外的成员，除非获得您的许可。所有的研究成员和研

究申办方都被要求对您的身份保密。为确保研究按照规定进行，必要时，政府管理部门或伦理审查委员会的成员按规定可以在研究单位查阅您的个人资料。

如果这项研究结果发表时，研究者也需要对保密方面进行承诺。

**费用和补偿：**本研究不会影响您原有的治疗方案及治疗费用。

**自由退出：**

作为受试者，您可随时了解与本研究有关的信息资料和研究进展，自愿决定（继续）参加还是不（继续）参加。参加后，无论是否发生伤害，是否严重，您可以选择在任何时候不需要任何理由通知研究者要求退出研究，您的数据将不纳入研究结果，您的任何医疗待遇与权益不会因此而受到影响。如果继续参加研究，会对您造成严重的伤害，研究者也将会终止研究的进行。

但在参加研究期间，请您提供有关自身病史和当前身体状况的真实情况；告诉研究医生自己在本次研究期间所出现的任何不适；不得服用受限制的药物、食物等；告诉研究医生自己在最近是否曾参与其他研究，或目前正参与其他研究。如果因为您没有遵守研究计划，或者发生了与研究相关的损伤或者有任何其它原因，研究医师可以终止您继续参与本项研究。

**联系方式：**

如果您有与本研究有关的问题，或您在研究过程中发生了任何不适与损伤，或有关于本项研究参加者权益方面的问题，您可以与钱昆，**电话** 18623184180。【项目组的联系人必须为有资质的本院医生】

**试验后利益分享：**

本研究为理论性成果，暂无利益分享。

**知情同意签字：**

我已经阅读了本知情同意书，并且我的医生 钱昆 已经将此次临床试验的目的、内容、风险和受益情况向我作了详细的解释说明，对我询问的所有问题也给予了解答，我对此项临床研究已经了解，我自愿参加本项研究。

受试者签名：陈亚东

日期：2024 年 03 月 27 日

研究者签名：钱昆

日期：2024 年 03 月 27 日

本研究为理论性成果，暂无利益分享。

**知情同意签字：**

我已经阅读了本知情同意书，并且我的医生钱昆已经将此次临床试验的目的、内容、风险和受益情况向我作了详细的解释说明，对我询问的所有问题也给予了解答，我对此项临床研究已经了解，我自愿参加本项研究。

受试者签名：杨朝华

日期：2024年 02月 07日

研究者签名：钱昆

日期：2024年 02月 07日

本研究为理论性成果，暂无利益分享。

**知情同意签字：**

我已经阅读了本知情同意书，并且我的医生________已经将此次临床试验的目的、内容、风险和受益情况向我作了详细的解释说明，对我询问的所有问题也给予了解答，我对此项临床研究已经了解，我自愿参加本项研究。

受试者签名：________

日期：2024年 2月 16日

研究者签名：________

日期:2024年 2月 17日
