# Supplementary material for: Mechanistic insights into VSIR-mediated AXL regulation and gastric cancer proliferation
Source: iScience. 2026 Sep 18;29(10):117580. doi: 10.1016/j.isci.2026.117580 (PMC13626921; doi:10.1016/j.isci.2026.117580)
Supplement: Supplementary file 3 [file mmc3.pdf]

重庆医科大学附属第一医院医学研究伦理审查委员会

伦理审查批件

批件号：2026 年科研伦审（2026-0273-01）

<table>
<tr><td>药物/器械名称</td><td colspan="3">NA</td></tr>
<tr><td>项目名称</td><td colspan="3">VSIR介导的AXL调控与胃癌增殖的机制研究</td></tr>
<tr><td>试验分类</td><td colspan="3">☐药物临床试验 ☐医疗器械临床试验 ■临床研究 ☐其他</td></tr>
<tr><td>科室/项目负责人</td><td>胃肠外科 钱昆</td><td>组长单位</td><td>单中心</td></tr>
<tr><td>申请单位/项目来源</td><td colspan="3">自拟课题</td></tr>
<tr><td rowspan="2">审查情况</td><td colspan="3">☐ 会议审查 审查日期：
伦理委员共 人，实到 人，回避 人（**回避），参与投票 人。
同意 票，作必要的修正后同意 票，作必要的修正后重审 票，不同意 票。</td></tr>
<tr><td colspan="3">■ 快速审查（初始审查）（主审委员：张军、李俊龙 ） 审查日期：2026.04.15</td></tr>
<tr><td>审查文件</td><td colspan="3">详见附件1：《重庆医科大学附属第一医院医学研究伦理审查文件清单》（盖章页）</td></tr>
<tr><td rowspan="3">审查意见</td><td colspan="3">评审决议：
该项目资料齐备，研究者资格符合要求，研究方案适当，免除知情同意书申请符合伦理要求，同意该项目在我院进行临床研究。</td></tr>
<tr><td colspan="3">审查频率为伦理首次批准之日起：☐6 个月 ■12 个月 ☐不变 ☐其他</td></tr>
<tr><td colspan="3">主任委员或被授权的副主任委员（签名）：[签名]
重庆医科大学附属第一医院医学研究伦理审查委员会（盖章）
日期：2026.4.15</td></tr>
</table>

注意事项：（请仔细阅读）

1. 研究中研究者须严格遵守 NMPA、GCP 和《赫尔辛基宣言》的相关规定。
2. 已批准项目须遵循本伦理委员会批准的方案执行，对已批准的临床研究方案、知情同意书等材料的任何修改及主要研究者更换等，须及时上报本伦理审查委员会重新审查，并须获得批准后执行。
3. 在本院发生的严重不良事件，须 24 小时内报告本伦理审查委员会。
4. 发现违反方案情况须及时报告。
5. 暂停/提前终止临床研究，请及时通知伦理审查委员会。
6. 本报告自签署之日起一年内有效，研究进行过程中将接受本伦理委员会的持续审查，无论试验开始与否，请在持续日到期前一个月提出持续审查申请。
7. 完成临床研究，须提交结题报告供伦理审查委员会审查。
8. 凡涉及中国人类遗传资源采集标本、收集数据等研究项目，必须获得中国人类遗传资源管理办公室批准后方可在本中心开展研究。
9. 凡经本伦理审查委员会批准的研究项目在实施前，申请人应按相关规定在国家卫健委、药审中心等的临床研究登记备案信息系统平台登记研究项目相关信息。
10. 重庆医科大学附属第一医院医学研究伦理审查委员会联系电话：023-89011876，地址：重庆市渝中区袁家岗友谊路 1 号。

声明：本伦理委员会的组成及工作程序符合《赫尔辛基宣言》、《涉及人的生命科学和医学研究伦理审查办法》、《科技伦理审查办法（试行）》、ICH-GCP、中国 GCP 等国际伦理原则及我国的相关法律法规和规章制度。

附件 1：重庆医科大学附属第一医院医学研究伦理审查文件清单

附件 1：批件号 2026 年科研伦审（2026-0273-01）审查文件清单

1. 科学性审查意见
2. 研究方案 （1.0 版，2026.02.01）
3. 免除知情同意书申请
4. 主要研究者简历及资质
5. 研究者利益冲突声明

重庆医科大学附属第一医院伦理委员会

VISR介导的AXL调节与胃癌增殖的机制研究

机构名称：　　重庆医科大学附属第一医院

版本号：　　V 1.0

版本日期：　　2026年2月1日

主要研究者：　　钱昆

方案签字页

我将根据中国 GCP 及《医疗卫生机构开展研究者发起的临床研究管理办法》等的规定，认真履行研究者职责，亲自参加或直接指导本临床研究。我熟悉本项临床研究的研究方案（版本号：[V 1.0]；版本日期：[2026-02-01]）。我同意按照中国法律、赫尔辛基宣言、中国 GCP 以及此研究方案履行相关的职责。我将负责做出与临床相关的医学决定，保证患者在研究期间出现不良事件时能及时得到适当的治疗，并按照国家有关规定要求记录和报告这些不良事件。我保证将数据真实、准确、完整、及时地记录。我将接受临床研究管理部质量管理员的质量监查，确保临床研究的质量。我承诺对患者个人信息和相关事宜进行保密。我同意应要求公开与临床研究有关的开支，同意禁止与本研究相关的商业、经济行为。

主要研究者姓名：

主要研究者（签字）：____________　　签字日期：2026 年 3 月 3 日

一、研究的背景和目的

胃癌是我国高发恶性肿瘤，增殖失控是其恶性进展与预后不良的核心环节。AXL 作为 TAM 家族受体酪氨酸激酶，在胃癌中异常高表达，其配体 Gas6 结合后触发自身磷酸化，持续激活下游促增殖信号，与肿瘤分期、侵袭转移及不良预后显著相关[1,2,9]。现有研究提示 AXL 通过 PI3K/Akt、ERK/MAPK、FAK/Src 等通路调控胃癌细胞增殖[7,8]，但上游调控、关键效应分子、转录调控及肿瘤微环境交互的具体机制仍不明确，制约靶向干预转化。

本研究旨在阐明 AXL 介导胃癌增殖的分子机制：①检测临床组织中 AXL、p-AXL 及下游增殖信号表达，分析与临床病理及预后的相关性；②构建敲低/过表达 AXL 细胞模型，明确其对胃癌细胞增殖、克隆形成、周期进程的影响；③解析 Gas6/AXL 激活 PI3K/Akt、ERK/MAPK 等通路的级联反应，鉴定关键节点分子；④探索 AXL 的上游调控（如 miRNA、甲基化、CAF 微环境）与下游转录调控网络[2,6]；⑤体内验证 AXL 抑制对移植瘤生长的作用[9]。

本研究将明确 AXL 驱动胃癌增殖的核心通路与分子靶点，为胃癌提供新型预后标志物与精准治疗靶点[1,5]，为开发 AXL 靶向药物、克服增殖异常与耐药奠定理论与实验基础[7,9]。

二、研究的设计和方法

研究对象：本研究为一项回顾性研究，拟于病理科收集重庆医科大学附属第一医院 2021.01-2025.01 期间既往胃癌病人手术病理切片，预计 115 例。

本研究以新辅助治疗后病理切片提示肿瘤退缩程度为主要结局指标，根据文献验得：

对照组有效率 p1=45%，试验组预期 p_2=73%，

取 $\alpha=0.05$，$1-\beta=0.80$，采用两独立样本率比较公式计算：

$$n=\frac{(1.96+0.84)^2[p_1(1-p_1)+p_2(1-p_2)]}{(p_1-p_2)^2}$$

计算得每组需 n=98 例，考虑 10%~20%失访，最终每组纳入 115 例。

纳入标准：2021.01-2025.01 我院确诊胃恶性肿瘤行术前新辅助化疗并进行根治性手术的患者手术病理切片

研究内容

1.1.1 VSIR 以脂多糖依赖的方式促进 AXL mRNA 表达

为评估 AXL 在胃癌中的临床相关性，我们通过 GEPIA2 平台从癌症基因组图谱胃腺癌（TCGA-STAD）队列中检索了公开可用的数据。AXL 表达水平随着肿瘤分期的进展而逐渐升高，在 IV 期显著高于 I 期。此外，基于 TCGA-STAD 数据的 Kaplan-Meier 生存分析表明，AXL 高表达患者的总体生存率显著低于低表达患者[1]。这些公开可用的数据表明 AXL 上调与胃癌的疾病进展和不良预后之间存在很强的关联。与我们从 TCGA-STAD 数据集观察到的 AXL 在晚

期胃癌中上调且与不良预后相关这一结果一致，我们试图探究 AXL 过表达的调控机制。转录组测序显示，在 VSIR 过表达联合 LPS 刺激的条件下，AXL mRNA 显著升高。为了验证这一发现，我们在 HGC-27 细胞中进行了 RT-qPCR 检测，结果证实，在没有脂多糖（LPS）的情况下，VSIR 过表达未能诱导 AXL mRNA 的表达；然而，在 LPS 处理后，VSIR 显著提高了 AXL 转录水平。蛋白质印迹分析进一步在蛋白质水平上证实了这些结果。为了确定 VSIR 是否是 LPS 诱导 AXL 表达所必需的，我们设计了针对 VSIR 的 siRNA，并在 LPS 刺激前将其转染到 HGC-27 细胞中。VSIR 的敲低有效地消除了 LPS 介导的 AXL 表达诱导，表明 VSIR 是胃癌细胞对 LPS 作出反应上调 AXL 所必需的。总之，这些数据表明 VSIR 特异性地在由 LPS 模拟的炎症条件下促进 AXL 表达，为先天免疫信号传导与 AXL 驱动的胃癌进展之间的潜在机制联系提供了证据[1,2]。

1.1.2 STAT3 直接与 AXL 增强子结合并驱动其转录激活

为了确定 AXL 的潜在转录调节因子，我们使用多个转录因子结合位点数据库（hTFtarget、CHEA、FIMO_JASPAR 和 ENCODE）进行了计算预测。通过维恩图分析发现，STAT3 和 EGR1 这两种转录因子在所有四个数据集中均被预测为与 AXL 基因的调控区域结合，这表明它们可能在 AXL 表达调控中发挥作用[2,7]。为了进一步验证这些候选因子的功能相关性，我们使用 Cistrome 数据库检查了 AXL 位点的染色质可及性和组蛋白修饰图谱。我们观察到，在 AXL 基因上游的一个增强子区域内，STAT3 结合信号显著富集，与活跃的组蛋白标记 H3K27ac（与增强子激活相关）和 H3K4me1（潜在或活跃增强子的标志）重合。相比之下，IGF1R 没有检测到此类调控特征。这些数据表明，STAT3 很可能结合在 AXL 附近的活性增强子元件上，并可能直接参与其转录调控。基于这些发现，我们选择 STAT3 作为候选调节因子进行进一步研究。为了实验验证 STAT3 调控 AXL 表达的生物信息学预测，我们首先在 HGC-27 细胞中进行了染色质免疫沉淀后定量 PCR（ChIP-qPCR）。使用跨越 CistromeDB 中鉴定出的 AXL 基因上游假定增强子区域的引物，我们检测到与对照 IgG 相比，该位点 STAT3 结合显著富集，这证实了 STAT3 与 AXL 调控区域的直接物理相互作用[6,7]。

接下来，我们将包含预测的 STAT3 结合基序的相同增强子片段克隆到荧光素酶报告载体中。将此构建体与 JAK1 和 STAT3 表达质粒共转染显著增加了荧光素酶活性。最后，为了评估 AXL 对 STAT3 的功能依赖性，我们使用 siRNA 敲低了 HGC-27 细胞中的 STAT3。RT-qPCR 和 Western blot 分析均显示，STAT3 敲低显著降低了 AXL 的 mRNA 和蛋白水平。这些数据共同表明，STAT3 是一种转录因子，可直接与 AXL 上游的增强子元件结合，并且对于胃癌细胞中 AXL 的基础表达和诱导表达至关重要[2,7]。

1.1.3 STAT3 对脂多糖诱导的 AXL 上调至关重要

为了研究 STAT3 在介导 LPS 诱导的 AXL 表达中的生理相关性，我们建立了一个髓系特异

性 STAT3 条件性敲除（STAT3-cko）小鼠模型。将 STAT3fl/fl 小鼠与 LyzM-Cre 转基因小鼠杂交后，可在髓系细胞中特异性地高效缺失 STAT3，这一点经基因分型 PCR 证实。然后，我们从野生型（WT）和 STAT3-cko 小鼠中分离骨髓来源的巨噬细胞（BMDMs），并用 LPS 刺激。Western blot 分析显示，虽然 LPS 处理显著增加 WT BMDMs 中的 AXL 蛋白水平，但这种诱导作用在 STAT3-cko BMDMs 中完全被消除，表明在原代髓系细胞中，LPS 介导的 AXL 上调需要 STAT3[7]。为了在人胃癌细胞中证实这些发现，我们在 HGC-27 细胞中进行了 siRNA 介导的 STAT3 敲低。与结果一致的是，在基础和 LPS 刺激条件下，LPS 诱导的 AXL 表达在 STAT3 敲除后显著降低。总之，这些数据表明，在髓系细胞和胃癌细胞中，STAT3 是 LPS 诱导 AXL 表达的关键转录介质[2,7]。

1.1.4 在 LPS 刺激下，VSIR 通过促进 IL-6 的产生和 STAT3 的活化来促进 AXL mRNA 的表达

热图分析和 RT-qPCR 结果显示，在 LPS 刺激下，过表达 VSIR 显著增加了 IL-6 mRNA 水平的表达。同时，VSIR 过表达诱导了 STAT3 的磷酸化，表明 STAT3 信号通路被激活[7]。这些数据表明，VSIR 通过增强 IL-6-STAT3 信号级联来上调 AXL mRNA 的表达[2,6]。

1.1.5 VSIR 促进 AXL 蛋白稳定性

数据表明，VSIR 过表达增强了 LPS 诱导的 IL-6-STAT3 信号通路和上调 AXL 的表达[2,7]。在这里，我们进一步表明 VSIR 直接与 AXL 相互作用，导致 AXL 蛋白水平的稳定性增加。Western blot 分析显示，在 VSIR-HA 过表达后，AXL 蛋白表达呈剂量依赖性增加（图 5A），而免疫共沉淀实验证实了 VSIR 和 AXL 之间的物理相互作用（图 5B）。免疫荧光染色进一步支持它们在细胞区室中的共定位(图 5C)。这些发现表明，VSIR 不仅在转录水平调节 AXL 的表达，而且通过直接结合稳定 AXL 蛋白，从而放大下游炎症信号通路，如 IL-6-STAT3[9]。

1.1.6 VSIR 通过 AXL 依赖方式促进胃癌细胞增殖

为了研究 VSIR 和 AXL 在胃癌中的临床相关性，我们对组织样本进行了多重免疫荧光染色，包括 HER2 阳性治疗耐药（HER2⁺/TR）， HER2 阳性 treatment-naïve (HER2⁺/TN)， HER2 阴性（HER2⁻）和正常胃组织。我们的结果显示，与正常黏膜和 HER2 阴性样本相比，VSIR 和 AXL 在 HER2 阳性肿瘤中的表达水平显著升高[1,5]。定量分析证实，在 HER2 阳性耐药肿瘤中，VSIR+AXL+HER2+细胞的百分比显著高于其他组，表明与疾病进展和治疗耐药相关的强共表达模式[2,9]。既往研究表明，AXL 作为一种受体酪氨酸激酶，通过激活 PI3K/AKT、MAPK/ERK 等下游信号通路，在促进肿瘤细胞增殖、生存、迁移和治疗抵抗中发挥关键作用[7,8]。与这些发现一致的是，我们观察到在胃癌细胞中，VSIR 过表达显著增加了 Ki67 的 mRNA 和蛋白水平，Ki67 是细胞增殖的一个公认的标志物。重要的是，这种增殖作用在 AXL 抑制剂治疗后被显著抑制，这表明 VSIR 通过 AXL 依赖的机制发挥其促增殖功能[9]。综上所述，这些结果表明，VSIR 增强 AXL 的稳定性和表达，导致增殖信号的激活和 Ki67 的上调。VSIR-AXL 轴促进肿瘤生长，可能是一个潜在的治疗靶点，特别是在标准治疗耐药的 HER2 阳性胃癌中[1,2]。

1.1.7 VSIR 通过 AXL 依赖的方式促进体内肿瘤生长

利用稳定过表达 VSIR 的 HGC-27 胃癌细胞（OE-VSIR）和空载体对照（NC）建立裸鼠皮下移植瘤模型，评价 VSIR 在体内胃癌进展中的作用[9]。将荷瘤小鼠随机分为 5 个治疗组（n = 5）：(1)阴性对照组（NC）；(2) OE-VSIR;(3) OE-VSIR + HER2 抑制剂；(4) OE-VSIR + AXL 抑制剂；(5) OE-VSIR + HER2/AXL 双抑制剂。治疗 30 d 后，取肿瘤进行形态学和分子生物学分析。结果显示，与 NC 组相比，OE-VSIR 显著促进肿瘤生长，表现为肿瘤体积和重量增加。值得注意的是，单独使用 AXL 抑制剂显著抑制了 VSIR 诱导的肿瘤生长，使肿瘤大小和重量降低到与对照组相当或低于对照组的水平[7,9]。相比之下，单独抑制 HER2 仅对肿瘤生长产生小幅抑制作用。引人注目的是，HER2 和 AXL 的联合阻断产生了最有效的抗肿瘤作用，几乎取消了 VSIR 的促肿瘤活性[1,5]。为了评估体内增殖，我们在肿瘤切片中通过免疫组织化学（IHC）评估了 Ki-67 的表达。与肿瘤生长数据一致，Ki-67 染色强度在 OE-VSIR 组最高，AXL 抑制后明显降低[9]。使用 HALO™软件对 Ki-67 免疫反应性进行的定量分析证实了这些观察结果，显示 AXL 抑制剂组和双抑制剂组的积分光密度（IOD）显著降低。这些结果表明，VSIR 主要通过 AXL 依赖的机制促进体内胃癌的生长，并且联合靶向 AXL 和 HER2 可协同抑制 VSIR 驱动的肿瘤发生[1,9]。

2.2 研究方法

2.2.1 临床样本检测

入组患者和标本获取：回顾性收集重庆医科大学附属第一医院胃肠外科手术切除的 HER2 阳性治疗耐药（$HER2^+$ /TR）、HER2 阳性初治($HER2^+$ /TN)、HER2 阴性（$HER2^-$ ）和正常胃组织。全部病例均通过临床、影像学、病理诊断，术前未进行辅助治疗，临床病理及随访资料齐全。

2.2.2 多重免疫荧光

根据多重免疫荧光实验操作正规流程，将包埋石蜡组织制备成切片置于载玻片，在样本上滴加封闭液（如 BSA 或正常血清），以防止非特异性结合。封闭后，加入第一抗体进行孵育，在 4° C 下过夜，使用荧光标记的二抗进行染色，每种抗体对应一种荧光染料，室温下孵育 1 小时。在所有抗体孵育完成后，使用 DAPI 对细胞核进行染色，以便于观察细胞核的位置和分布。使用荧光显微镜或共聚焦显微镜进行成像，并通过图像处理软件 Image J 或 Fiji 软件对信号进行量化分析。

2.2.3 小鼠模型构建

所有动物程序均经恩施州土家族苗族自治州中心医院机构动物保护使用委员会批准（伦理批准文号：202412001)，并按照机构标准操作程序（SOP）进行已确认正确。采用 4 ~ 5 周龄雌性 BALB/c 裸鼠（武汉美 halic 生物技术有限公司，中国）建立胃癌皮下移植瘤。培养 HGC-27

和 VSIR 过表达稳定细胞株，用胰蛋白酶分离，在 4 ℃以 1000 rpm 离心 5 min。将细胞颗粒重新悬浮在含有 Matrigel（100 μL/只）的冰冷 RPMI-1640 培养基中，并调整为每只小鼠 5×10^6个细胞。小鼠被随机分为 5 组（n = 6 /组）。所有注射均在紫外线消毒的生物安全柜中进行。将细胞植入前肢皮下，轻轻按压注射部位 2 min，以减少渗漏。根据实验组进行标记。口服给药于接种后 24 h 开始，并持续 14 天：HER2 抑制剂（200 mg/kg/d）溶于 10% DMSO / 90%（20% SBE-β-CD 生理盐水溶液）；AXL 抑制剂（100 mg/kg/天）溶于载体（7.14% Cremophor EL，2.85%乙醇，90%盐水，0.1 M HCl 调 pH）。第 2 天肉眼观察证实肿瘤形成，从第 10 天开始每周使用数字卡尺测量肿瘤大小。肿瘤体积计算公式为：体积= 1 / 2 ×（宽度^2×长度）。如果肿瘤直径超过 1.5 cm，则在第 30 天或之前处死小鼠。切除的肿瘤固定在 4%多聚甲醛中进行免疫组化分析。

2.2.4 统计学分析

所有数据以均数±标准差（s.d.）表示。除非在相应的图例中另有说明，使用双侧非配对 t 检验评估统计学显著性。以 $P\leq0.05$ 为差异有统计学意义的阈值。每个图例中均明确说明了样本量(n)，表示每个实验组的小鼠数量。对于涉及两组以上比较的实验，采用单向方差分析后的 Dunnett 检验或双向方差分析，详细信息见相关图例。在这些分析中，$P\leq0.05$ 被认为是差异具有统计学意义。

三、研究的风险

基于回顾性研究的局限性，可能存在选择偏倚和信息偏倚。我们将采用 PSM 进行统计学调整，以尽可能减少偏倚。

患信息泄露的风险。我们将严格遵守伦理原则，对数据进行脱敏处理，确保无法识别患者身份。

四、受试者招募

该项目为回顾性研究，只需从医院电子病历系统里面调数据进行分析，并使用既往患者人体组织病理学切片，无需研究参与者招募。

五、知情同意过程

该项目为回顾性研究，只需从医院电子病历系统里面调数据进行分析，并使用既往患者人体组织病理学切片，已提交免知情同意书申请。

六、费用和补偿

该项目为回顾性研究，只需从医院电子病历系统里面调数据进行分析，并使用既往患者人体组织病理学切片，不会增加研究参与者的经济负担和身体损伤。研究过程数据会做脱敏

处理，极力保障研究参与者的数据安全和隐私安全。该项目无费用无补偿。

七、数据的获得和质控

(1)数据获得途径:数据来源包括患者的病历记录、实验室检测结果以及研究过程中采集的问卷和其他临床信息以及对既往患者人体组织病理学切片的观察。所有数据均由研究团队通过标准化流程采集、并记录在加密的电子数据库中。

(2)质量控制措施:采用双人核对录入制度，确保数据录入的准确性;定期对数据进行完整性和一致性检查，发现异常数据时及时追溯并修正;数据管理团队负责监督数据采集和存储过程，确保符合伦理和法规要求。

八、有关研究参与者的信息和保密

(1)数据使用范围:研究参与者的个人数据，仅用于本研究目的、不会用于其他用途;所有样品和数据均匿名化存储，并以唯一编码标识，避免个人身份信息泄露。

(2)数据访问权限:数据访问权限仅限于项目组成员、数据分析人员以及伦理委员会授权的审查人员;研究结果公开发表时，将以匿名形式呈现，不涉及研究参与者的任何个人信息。

(3)数据保密措施:数据存储在符合安全标准的加密数据库中，并由专人管理;涉及敏感信息的纸质文件存放在上锁的柜体内，非授权人员不得接触。

九、成果分享

研究结束后，所有相关成果将在科学期刊上发表，并可能在学术会议上进行报告;向全球科研人员分享研究发现，推动该领域的学术交流与知识更新。

参考文献

[1] Ho H, Cheng C Y, Hu et al. Phosphorylated AXL expression correlates with survival in gastric cancer patients, and targeting GAS6/AXL restores tumor immunogenicity and improves immunotherapy response[J]. J Clin Med,2024.

[2] Kim T H, Lee D, Oh et al. Cancer-associated fibroblast-derived GAS6 increases chemotherapy resistance in gastric cancer via the AXL/STAT3/ABCC1 axis[J]. Life Sciences,2023.

[3] Kim T H, Lee D, Oh et al. Loss of Die1/VISTA expression is an epigenetically regulated recurrent event in gastric cancer[J]. Br J Cancer,2026.

[4] Oliveira P, Carvalho J, et al. Inhibition of the STAT3-ferroptosis negative regulatory axis

suppresses tumor growth and alleviates chemotherapy resistance[J]. Sci Rep,2016.

[5] Ouyang S, Li H, Lou et al. Phosphoproteomic subtypes of gastric cancer reveal dynamic transitions to guide chemotherapy and targeted therapy[J]. Redox Biol,2022.

[6] Shoji H, Hirano H, No et al. Inhibition of the IL-6/STAT3 axis and AXL/Tyro3 receptor tyrosine kinases overcomes paclitaxel resistance[J]. Cell Rep,2024.

[7] Suh Y A, Jo S Y, Lee et al. Apigenin modulates drug resistance by targeting AXL and Tyro3[J]. Int J Oncol,2015.

[8] Tang Y, Zang H, Wen et al. AXL as a drug resistance regulator and therapeutic target in cancer[J]. J Exp Clin Cancer Res,2023.

[9] Tang Y, Zang H, Wen et al. AXL as a drug resistance regulator and therapeutic target in

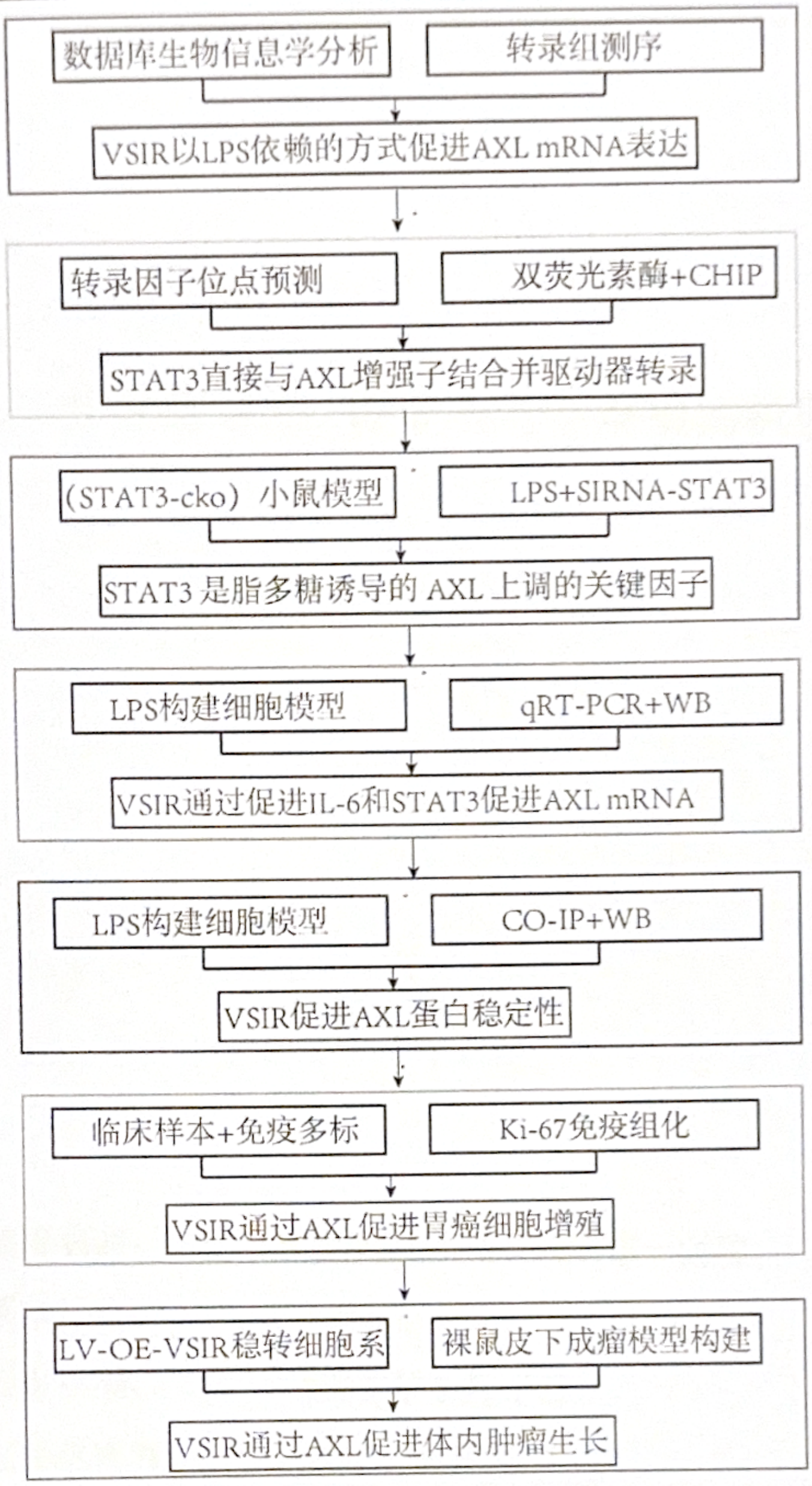

cancer[J]. J Exp Clin Cancer Res,2023.

关于将向伦理委员会申请免除知情同意的承诺书

项目名称	VISR 介导的 AXL 调节与胃癌增殖的机制研究		
申请科室	胃肠外科	项目负责人	钱昆
项目联系人	钱昆	电话号码	186 23184180

向伦理委员会申请免除知情同意，须符合以下免除知情同意的全部条件：	
☑	本研究使用的病历或相关数据是以往诊疗中获取的。
☑	涉及使用生物样本的研究，研究使用的生物样本来源于我院生物样本库，且样本使用获得捐赠者的泛知情同意。
☑	本研究对研究参与者的风险不大于最小风险[1]。
☑	免除知情同意不会对研究参与者的权利和健康产生不利的影响。
☑	研究参与者的隐私和个人身份得到保护。
☑	利用可识别身份信息的人体材料或者数据进行研究，已无法找到研究参与者，且研究项目不涉及个人隐私和商业利益的。

研究承诺：本研究项目不涉及个人隐私和商业利益，样本及相关信息仅用于本项目研究。
我们承诺将向伦理委员会提交免除知情同意申请。

项目负责人签名：钱昆　　　　日期：2026年3月5日

[1] 最小风险（Minimal Risk):指试验中预期风险的可能性和程度不大于日常生活、或进行常规体格检查或心理测试的风险。

知情同意书

研究背景介绍（简写）：胃癌是威胁人类健康的主要消化道肿瘤之一。胃癌的形成、侵袭和转移与肿瘤细胞免疫逃逸关联。对胃癌原发灶和癌旁正常组织进行单细胞测序。单细胞测序结果表明：VSIR 基因在胃癌组织中明显升高，在巨噬细胞和单核细胞中的表达量异常升高，提示VSIR 基因在胃癌的形成过程中发挥了重要作用。本课题拟在单细胞测序的基础上，分析 VSIR 在胃癌形成中如何使胃癌细胞产生免疫逃逸的作用机制。然后通过体内外实验及蛋白质 4D 技术研究VSIR在胃癌形成过程中的机制。

尊敬的患者（受试者）：

您好！

由于您是胃癌患者，需要进行手术治疗，您将被邀请参加一项由重庆医科大学附属第一医院胃肠外科钱昆教授主持的研究。这是为探索VSIR 在胃癌形成过程中的机制而进行一项研究，它将历时 3 年时间。此项目由重庆市科技厅重大项目资助进行。

本知情同意书提供给您一些信息以帮助您决定是否参加此项临床研究。您参加本项研究是自愿的。本次研究已通过本研究机构伦理审查委员会审查。如果你同意加入此项研究，请看下列说明：

请您仔细阅读，如有任何疑问请向负责该项研究的研究者提出。

研究目的：（背景意义—简写）

胃癌是威胁人类健康的主要消化道肿瘤之一。胃癌的形成、侵袭和转移与肿瘤细胞免疫逃逸关联。对胃癌原发灶和癌旁正常组织进行单细胞测序。单细胞测序结果表明：VSIR 基因在胃癌组织中明显升

高，在巨噬细胞和单核细胞中的表达量异常升高，提示VSIR 基因在胃癌的形成过程中发挥了重要作用。本课题拟在单细胞测序的基础上，分析 VSIR 在胃癌形成中如何使胃癌细胞产生免疫逃逸的作用机制。然后通过体内外实验及蛋白质 4D 技术研究VSIR 在胃癌形成过程中的机制。

研究过程和方法（简写）:

如果您同意参与这项研究，我们将对每位受试者进行编号，建立病历档案。在研究过程中我们需要采集一些您的标本，将由专业人员为您取样，我们将从您手术切下的标本上取一小块组织进行研究，您的生物样品仅用于本次研究。

研究可能的受益:

经过本项目的研究，寻找到关于胃癌新的免疫检查点，然后与相关药企合作，实现对胃癌的免疫靶向治疗，为胃癌患者带来巨大福音，造福社会、造福人类，同时也可以实现巨大的经济价值。

研究风险与不适: 在您的手术标本上取一小块组织不会对您后续的诊断和治疗造成任何影响，也不会增加您的治疗费用。

其他治疗干预方式:

您除了参加此研究外，无其他的干预或治疗措施。

隐私问题:（过程中的隐私保护和结果发表的隐私保护）

如果您决定参加本项研究，您参加试验及在试验中的个人资料均属保密。对于您来说，所有的信息将是保密的。您的病理标本将以研究编号数字而非您的姓名加以标识。可以识别您身份的信息将不会透露给研究小组以外的成员，除非获得您的许可。所有的研究成员和研

究申办方都被要求对您的身份保密。为确保研究按照规定进行，必要时，政府管理部门或伦理审查委员会的成员按规定可以在研究单位查阅您的个人资料。

如果这项研究结果发表时，研究者也需要对保密方面进行承诺。

费用和补偿：本研究不会影响您原有的治疗方案及治疗费用。

自由退出：

作为受试者，您可随时了解与本研究有关的信息资料和研究进展，自愿决定（继续）参加还是不（继续）参加。参加后，无论是否发生伤害，是否严重，您可以选择在任何时候不需要任何理由通知研究者要求退出研究，您的数据将不纳入研究结果，您的任何医疗待遇与权益不会因此而受到影响。如果继续参加研究，会对您造成严重的伤害，研究者也将会终止研究的进行。

但在参加研究期间，请您提供有关自身病史和当前身体状况的真实情况；告诉研究医生自己在本次研究期间所出现的任何不适；不得服用受限制的药物、食物等；告诉研究医生自己在最近是否曾参与其他研究，或目前正参与其他研究。如果因为您没有遵守研究计划，或者发生了与研究相关的损伤或者有任何其它原因，研究医师可以终止您继续参与本项研究。

联系方式：

如果您有与本研究有关的问题，或您在研究过程中发生了任何不适与损伤，或有关于本项研究参加者权益方面的问题，您可以与钱昆，**电话** 18623184180。【项目组的联系人必须为有资质的本院医生】

试验后利益分享：

本研究为理论性成果，暂无利益分享。

知情同意签字：

我已经阅读了本知情同意书，并且我的医生 [illegible] 已经将此次临床试验的目的、内容、风险和受益情况向我作了详细的解释说明，对我询问的所有问题也给予了解答，我对此项临床研究已经了解，我自愿参加本项研究。

受试者签名：余云萍

日期：2026年 4月 19日

研究者签名：[illegible]

日期：2026 年 04月 19日

本研究为理论性成果，暂无利益分享。

知情同意签字：

我已经阅读了本知情同意书，并且我的医生______________已经将此次临床试验的目的、内容、风险和受益情况向我作了详细的解释说明，对我询问的所有问题也给予了解答，我对此项临床研究已经了解，我自愿参加本项研究。

受试者签名：潘庆生

日期：2026 年 4 月 20 日

研究者签名：

日期：2026 年 04 月 21 日

本研究为理论性成果，暂无利益分享。

知情同意签字：

我已经阅读了本知情同意书，并且我的医生钱昆________已经将此次临床试验的目的、内容、风险和受益情况向我作了详细的解释说明，对我询问的所有问题也给予了解答，我对此项临床研究已经了解，我自愿参加本项研究。

受试者签名：周道信

日期：2026 年 4 月 23 日

研究者签名：钱昆

日期：2026 年 04 月 23 日

本研究为理论性成果，暂无利益分享。

知情同意签字：

我已经阅读了本知情同意书，并且我的医生______已经将此次临床试验的目的、内容、风险和受益情况向我作了详细的解释说明，对我询问的所有问题也给予了解答，我对此项临床研究已经了解，我自愿参加本项研究。

受试者签名：______

日期：2026年 4 月 17 日

研究者签名：______

日期：2026年 4 月 17 日

本研究为理论性成果，暂无利益分享。

知情同意签字：

我已经阅读了本知情同意书，并且我的医生 [illegible] 已经将此次临床试验的目的、内容、风险和受益情况向我作了详细的解释说明，对我询问的所有问题也给予了解答，我对此项临床研究已经了解，我自愿参加本项研究。

受试者签名：[illegible]

日期：2026年 4月22日

研究者签名：[illegible]

日期：2026年 04月 22日

本研究为理论性成果，暂无利益分享。

知情同意签字：

我已经阅读了本知情同意书，并且我的医生__钱昆.____已经将此次临床试验的目的、内容、风险和受益情况向我作了详细的解释说明，对我询问的所有问题也给予了解答，我对此项临床研究已经了解，我自愿参加本项研究。

受试者签名：张小青

日期：2026年 4月20 日

研究者签名：钱昆

日期：2026年 04 月 20日

本研究为理论性成果，暂无利益分享。

知情同意签字：

我已经阅读了本知情同意书，并且我的医生________已经将此次临床试验的目的、内容、风险和受益情况向我作了详细的解释说明，对我询问的所有问题也给予了解答，我对此项临床研究已经了解，我自愿参加本项研究。

受试者签名：________

日期：2026年4月19日

研究者签名：________

日期：2026年04月19日

本研究为理论性成果，暂无利益分享。

知情同意签字：

我已经阅读了本知情同意书，并且我的医生 [illegible] 已经将此次临床试验的目的、内容、风险和受益情况向我作了详细的解释说明，对我询问的所有问题也给予了解答，我对此项临床研究已经了解，我自愿参加本项研究。

受试者签名：[illegible]

日期：2026年 4月 20日

研究者签名：[illegible]

日期：2026年 04月 20日

本研究为理论性成果，暂无利益分享。

知情同意签字：

我已经阅读了本知情同意书，并且我的医生________已经将此次临床试验的目的、内容、风险和受益情况向我作了详细的解释说明，对我询问的所有问题也给予了解答，我对此项临床研究已经了解，我自愿参加本项研究。

受试者签名：________

日期：2026年 04月 18日

研究者签名：________

日期：2026年 04月 18日
