# Supplementary material for: Mechanistic insights into VSIR-mediated AXL regulation and gastric cancer proliferation
Source: iScience. 2026 Sep 18;29(10):117580. doi: 10.1016/j.isci.2026.117580 (PMC13626921; doi:10.1016/j.isci.2026.117580)
Supplement: Supplementary file 4 [file mmc4.pdf]

CERTIFICATION

The Central Hospital of Enshi Tujia and Miao Autonomous Prefecture

Laboratory Animal Management Committee

No: 202412001 **DATA:2025.4.15**

This file is used to certify that the laboratory animals and animal experiment procedures are ethical in manuscript with the title of “Mechanistic Insights into VSIR-Mediated AXL Regulation and Gastric Cancer Proliferatione”.

The Laboratory Animal Management Committee of The Central Hospital of Enshi Tujia and Miao Autonomous Prefecture is responsible for overseeing the care and use of all vertebrate animals on laboratory, and composed of scientists, technicians, veterinarians, students, and members of the public. The committee reviews each proposal to use animals, and decides whether the project has valid scientific goals and whether it is ethical and humane. The committee insists the principle of the 3R’s—Replacement of animals whenever possible with alternatives such as computer models or cells; Reduction in the number of animals used; and Refinement of techniques to make them as humane as possible.

All procedures in the manuscript were reviewed in advance by the Laboratory Animal Management Committee of The Central Hospital of Enshi Tujia and Miao Autonomous Prefecture and also met the guidelines of the National Institutes of Health Guide for the Care and Use of Laboratory Animals.

Animal Strains	BALB/c-nu
Numbers	32
Principal Investigator	Guoquan Huang

恩施州中心医院动物实验中心
实验动物使用计划（Animal Using Protocol, AUP）

此栏信息由实验动物使用与管理委员会（IACUC）办公室填写，申请者请勿填写

IACUC 批准编号：202412001	修订号：______。
批准日期：2025.04.15	失效日期：2026.04.15 。

申请者须知

1. 为了节省纸张和时间，请申请者先提交电子版 AUP 至恩施州中心医院实验动物管理与使用委员会秘书处；
2. IACUC 秘书首先对收到的 AUP 进行形式审查，符合要求的将提交给 IACUC 审查，审核时间需要 **2** 周，之后会及时向申请者反馈结果；
3. 申请者根据审查反馈情况修改完善 AUP，在 IACUC 确认后打印**一式两份**，项目负责人在 AUP 上签字交一份到供应与服务办公室，自留一份。；
4. **本 AUP 表审核通过后，方可在服务办公室订购实验动物 ！！！**

一、基本信息（在合适的选择项前打"☑"）

1. 项目信息

☑初次申请　☐ 复审，原 **AUP** 号是：（　　　）

项目名称（中文）：关于 VSIR 介导的 AXL 调控及胃癌增殖的机制研究			
项目名称（English）：Mechanistic Insights into VSIR-Mediated AXL Regulation and Gastric Cancer Proliferation			
项目类型：☑ 科学研究　☐教学　☐其它：（　　）			
课题经费类型*：省级重大项目		课题经费编号：2022BCE024	
支持该项目的课题名称：基于单细胞测序技术研究 VSIR 基因在胃癌形成过程中促使胃癌细胞产生免疫逃逸的机制研究			
单位（院系）：恩施土家族苗族自治州中心医院			
项目负责人（PI）	中文：黄国全	职称/职务：副主任医师	
	拼音/English：Huang Guoquan		
Email：hgq0529@163.com		办公（实验室）电话：13469739238	
实验室地址：恩施土家族苗族自治州中心医院金龙院区			
动物实验负责人：谭勇		职称/职务：主管检验技师	
Email：tanyong951018@163.com		手机：18375757836	

*项目类型请填写：国家重大专项、支撑计划、国家/省/市/校级自然科学基金或其它类型基金

2. 实验人员信息

所列人员必须经过培训

姓名	实验动物从业人员上岗证编号	手机	Email	项目中的职责*	动物实验经历和资质
谭勇	TY20241043	18375757836	tanyong951018@163.com	实验设计、实验管理、主操作	获得湖北动物实验资格，通过实验室培训考试，学习动物模型建立，掌握动物实验基本操作

*职责包括：实验设计、实验管理、主操作、操作助手、麻醉、动物管理等

二、使用动物的目的和意义：

以一般非生物医学背景人员为对象，用通俗的语言描述研究目的，对人类、动物的健康或科学的贡献。（避免使用简写和缩写）

研究背景和前期工作：胃癌在全世界范围内高发。近年来，随着医疗水平的不断发展，肝癌的诊治水平也有所提高，但胃癌患者的早期诊断和治疗水平仍然未得到明显改善。肝癌的形成、侵袭和转移是一个涉及到多个基因参与、且共同作用的复杂过程。包括癌基因突变或异常过表达、抑癌基因失活、肿瘤细胞产生免疫逃逸等等。多种基因的参与促进了胃癌细胞的恶性表型，增加了胃癌细胞的异质性、诱导肿瘤血管生成、逃避宿主的免疫监视，促使肿瘤细胞产生免疫逃逸，最终导致胃癌细胞无限增殖并形成胃癌或是转移灶。目前全世界针对胃癌的靶向治疗研究尚欠。因此，需要我们深入探讨胃癌形成的分子机制，同时寻找到胃癌诊断和治疗的新靶向共计。

使用实验动物解决什么问题：

利用裸鼠构建人源性异种胃癌移植模型，研究 VSIR 影响胃癌的机制。

三、动物使用的理由和替代方法的检索（在所有适合的项目前打 ☑）

1. 通过何种方法得出实验所需的动物数量。

- ☑ 统计分析显示，实验计划的（动物）数量是最少的，符合统计学上对实验假设进行有效检验的要求。
- ☑ 实验将进行多个独立因素结果的对比，因此要求多组。
- ☑ 结果的检测或者被检测的现象是可变的，大样本量对于统计上有效的取样是必需的。
- ☑ 来自于对照组的差异预计是很小的。对于可靠地区分组间差异，大样本量是必需的。
- ☑ 实验具有技术上的困难，需要多次尝试以便从每一次实验中获得令人满意的数据。
- ☐ 其他（请解释）：

2. 在本实验中使用活体动物的理由是什么？

☐ 本研究要求对活体动物进行行为测量。
☑ 本研究要求对活体动物进行生物学检测或活体动物的组织样本取材。
☑ 在本实验中，计算机或者其他模型不能代替动物。
☐ 本研究不能做离体实验。
☐ 其他（请解释）：

3. 解释为什么你的研究中将要使用的动物种类是合理的。
☐ 本实验是以前使用该品种动物实验工作的直接延续。
☐ 本研究致力于扩展以前的从其它品种到这个品种的实验结果。
☐ 对于该品种动物很重要的生理/行为学现象，现在缺乏了解。
☐ 对于这个品种动物很重要的生理/行为学现象的相关内容比其他品种了解得更多。
☑ 这个品种的选择体现出了在能够使用的最低级的生物体和与人类的生理/行为最相近的模型系统中间最好的折衷方案。
☐ 对于计划开展的研究这个品种是最经济的。
☐其他（请解释）：

4. 在实验设计开始前寻找活体动物使用的替代方法。

至少选择 2 种查阅的数据库：<br>☑ PubMed ☑ Web of Scienc ☐ 知网 ☐ 万方 ☐ 维普 ☐ 其它数据库：（ ）
检索日期： （2025） 年（ 3）月（ 1）日<br>检索涵盖的年份： （ 2020） 年 至（2025）年
检索关键词（如：何种动物模型，非动物模型，疼痛，紧张，在体外，培养）：<br>胃癌、裸鼠、VSIR、体内
结论：是否有活体动物实验的替代方法： ☑ 没有 ☐ 有

四、所需实验动物信息（在所有适合的项目前打 ☑）

针对使用的每一种动物的品系填写每一栏。如果使用了 3 种以上或 3 个品系以上的动物，可在后面增加列。

内容	A	B	C
品种品系	balb/c		
性别	☑ 雌 F ☐雄 M<br>☐各半 Half F&M	☐雌 F ☐雄 M<br>☐各半 Half F&M	☐雌 F ☐雄 M<br>☐各半 Half F&M
年龄（日/周龄）或（和）体重（克/千克）	4 周龄		
动物数量	32		
动物来源	☑ 中心统一采购<br>☐ 国内外其它机构，机构名称：（ ）		

饲养环境	非感染性实验	☐ 平板架环境	☑IVC 环境
拟申请笼位数	5		
预计饲养时间	开始时间：（ 2025 年 4 月 18 日）	结束时间：（ 2025 年 07 月 30 日）	

五、实验设计和动物实验操作 （在合适的选择项前打“☑”）

1. 请详细描述整个实验从开始到结束的所有内容：包括所有实验流程、时间进度和具体实验方法

本实验计划使用裸鼠 32 只，4 周龄雌性。实验划分为 5 组，包含自然损耗在内，适应性饲养一周后。将 HGC-27 和 HGC-27-VSIR 过表达的稳定细胞系进行培养，用胰蛋白酶消化后，在 4°C 下以 1000 rpm 离心 5 分钟。细胞沉淀物重悬于冰冻的 RPMI-1640 培养基中，并加入 Matrigel（每注射 100 μL），调整至每只小鼠 5×10^6个细胞。根据实验组别对笼子进行标记，将小鼠随机分为五组（对照组：NC-HGC-27 和 OE-VSIR；药物处理组：OE-VSIR+HER2 抑制剂、OE-VSIR+AXL 抑制剂、OE-VSIR+HER2 抑制剂+AXL 抑制剂），每组 5 只小鼠（n=5/组）。所有注射操作均在紫外消毒的生物安全柜内进行。细胞植入前肢皮下，注射部位轻轻按压 2 分钟以减少渗漏。口服给药从接种后 24 小时开始，连续每日给药 14 天：HER2 抑制剂（曲妥珠单抗（伊尔比替尼）抑制剂，剂量方案：200 mg/kg/天；供应商：MedChemExpress (MCE)；货号：HY-16069；给药途径：口服，溶于 10% DMSO/90%（20% SBE-β-CD 在生理盐水中）溶液中）。试剂盒的推荐剂量与参考剂量（AXL(AXL-IN-13)抑制剂，给药方案：100 mg/kg/天；供应商：上海远业生物科技有限公司；货号：V38509；给药途径：静脉注射，溶剂为 7.14% Cremophor EL、2.85% 乙醇、90%生理盐水，含微量 0.1 M HCl）联合使用。试剂盒的推荐剂量与参考剂量[36]一致。第 2 天通过肉眼确认肿瘤形成，并从第 10 天起每周使用数字卡尺测量肿瘤尺寸。肿瘤体积计算公式为：体积 = ½ ×（宽² × 长）。若肿瘤直径超过 1.5 cm，于第 30 天或更早时进行安乐死处理。切除的肿瘤用 4%戊二醛固定，以便进行免疫组化分析。

注：请描述动物分组和每只动物的用途，动物数量应与动物信息表中的动物数相同；
涉及疾病模型的，要详细模型建立的原理和方法；
涉及手术的可简要描述，详细内容须在后面手术部分详细描述。

2. 动物标记方法 （禁止使用苦味酸！！！）

☑ 耳孔/耳标　　☐ 剪脚趾（7 天内的鼠适用）　　☑ 按笼盒区分

☐ 动物专用标记笔

2. 描述涉及动物给药或接种请写明药品名称、给药方式、使用浓度、剂量和频次；

物质名称	浓度	给药/接种剂量	体积 ml/kg	部位/途径	针头大小	频率
细胞悬液	5×10^6 /ml		4	腋下	25G	1 次
AXL 抑制剂	1g/ml	100 mg/kg/天	0.1ml/kg	口服		1 次/天
HER2 抑制剂	2g/ml	200 mg/kg/天	0.1ml/kg	口服	-	1 次/天

3. 描述涉及动物样本采集请写明样本名称、采集方式、采集量和频次；

样本名称	采集量	采集部位	采集方法（部位，针头规格等）	频率
肿瘤组织	完整肿瘤	皮下	用手术剪刀摘除	1

4. 是否在本中心外的设施中开展部分动物实验

☐ 是 ☑ 否， 如果是，请在下面描述实验内容、地点、环境标准、时间，转移防护措施，是否返回以及返回后的饲养区域

5. 是否存在单笼饲养

☐ 是 ☑ 否， 如果是，请在下面描述科学理由和如何增加环境丰富度的措施

6. 是否存在禁食禁水（手术前除外）

☐ 是 ☑ 否， 如果是，请在下面描述禁食禁水的科学理由、时间和如何保证动物不会出现危险状况

7. 自带饲料或使用特殊饲料

☐ 是 ☑ 否，如果是，请在下面描述饲料的厂家或来源、灭菌方法、提供灭菌凭证

8. 是否自带仪器设备进入动物房

☐ 是 ☑ 否，如果是，请在下面描述设备名称、体积、电源要求、使用时间、消毒措施

9. 是否存在长时间保定（长时间保定是指超过30分钟的物理保定）

☐ 是 ☑ 否，如果是，请在下面描述长时间保定的科学理由、方法、保定时间，如何保证动物的正常生理

六、是否存在外科手术 ☐是 ☑ 否， 如果是，请填下面手术相关内容：

（1）是否属于恢复性手术（恢复性：术后需要存活的） ☐是 ☐否

（2）是否需要对同一动物进行重复重大手术？ ☐是 ☐否：

如果是，请说明充分的科学理由：（ ）

（3）手术人员

手术主要操作人员	护理人员

（4）描述手术的详细方案和步骤

术前准备（不限于材料、器械、药品、动物和人员的准备）：<br>术中详细方案（不限于麻醉、术式、监护）：<br>术后护理（不限于保暖、饮食、止痛、抗感染、监护）：

七、紧张和疼痛的处理（在合适的选择项前打"☑"）

1. 请描述本实验可能对动物造成哪些不良后果和护理措施

	选择项
不良后果	☑呼吸困难 □进食困难 ☑体重快速下降 □神经紊乱 □行动困难<br>□严重感染 ☑肿瘤 □剧烈疼痛 □严重腹泻 □大量失血<br>□体温失调 □营养不良/脱水 □其它（ ）
护理措施	☑每天观察至少 2 次 ☑每周称重至少 2 次 □麻醉镇痛（详见麻醉镇痛部分）<br>□治疗措施（ ）

2. 参考美国农业部 USDA 分类标准，请选择疼痛级别：

☑ C 类：最小的，短暂的，一过性的或无疼痛或紧张

□ D 类：有疼痛和紧张，通过麻醉、镇痛、镇静等适当的方式缓解疼痛和紧张

□ E 类：有疼痛和紧张，但没有采取缓解的疼痛和紧张的措施

3. 麻醉剂的使用（

何种操作	麻醉剂名称	浓度	剂量	给药途径	给药频率
注射	三溴乙醇	1.25%	0.2ml/10g	腹腔注射	终端麻醉

注： 不推荐使用的麻醉剂：水合氯醛。

4. 镇痛剂的使用

何种操作	镇痛剂名称	浓度	剂量	给药途径	给药频率

5. 如果对动物造成疼痛，但没有使用麻醉剂和镇痛剂，请给出科学理由

科学理由：科学理由：皮下注射仅会造成一次性的轻微疼痛，不会对小鼠的身心健康造成不良影响。

参考文献：Zeng L, Zhou J, Wang X, Zhang Y, Wang M, Su P. Cadmium attenuates testosterone synthesis by promoting ferroptosis and blocking autophagosome-lysosome fusion. Free Radic Biol Med. 2021 Nov 20;176:176-188. doi: 10.1016/j.freeradbiomed.2021.09.028. Epub 2021 Oct 2. PMID: 34610361.

八、实验过程中动物安乐死标准和结束后动物的处置（在合适的选择项前打“☑”）

1. 请在下表中勾选至少 2 个可实施动物安乐死的指标

体重	☑体重迅速下降超过 20%
外观	☑被毛杂乱、无光泽　☐消瘦　☐严重腹水
行为	☑无法进食和饮水　☐昏迷　☐瘫痪<br>☐弓背蜷缩　☐颤抖　☐呼吸困难，发绀
症状	☑严重腹泻且治疗无效　☐严重脱水　☐感染严重且治疗无效
肿瘤相关	☑肿瘤直径超过 15mm（小鼠）和 28mm（大鼠）　☐肿瘤破溃和感染
其它标准	

2. 请描述实施安死术的方法

☐ CO_2 （大小鼠，不适合新生动物）

☑ 麻醉后脱颈椎（小鼠，200g 以下大鼠）：不推荐直接颈椎脱臼法，推荐 CO2 或异氟烷等麻醉后颈椎脱臼，如不先麻醉就行颈椎脱臼，请描述详细的科学理由：＿＿＿＿＿。

☐ 麻醉下灌流（大小鼠）：麻醉剂：＿＿剂量：＿＿＿途径：＿＿＿；

☐ 麻醉*后断头（大小鼠，需断头器）：麻醉剂：＿＿剂量：＿＿＿途径：＿＿＿；

☐ 麻醉后下放血（大小鼠）：麻醉剂：＿＿剂量：＿＿＿途径：＿＿＿；

☐ 过量注射戊巴比妥钠（兔、犬、猪、猴）：100mg/kg，静脉

☐ 其它安乐死的方法：

九、非药品级物质在动物体内的使用（在合适的选择项前打“☑”表示）

1. 使用情况（药品级物质，是指获得批准文号的可用于人体或动物体内的药品）

☑ 不使用　☐ 使用，如使用请继续填写

本计划中的非药品级物质属于：

☐ 化学试剂　☐ 生物制品（血清、抗体、蛋白）　☐细胞　☐ 其它（ ）

2.描述物质的相关信息

名称：	厂家/来源：
纯度：	等级：
配制方法：	
可溶性：	是否等渗： ☐ 是 ☐ 否
PH值：	
除菌方法：	保存条件：
给药途径：	

十、危险性物质的使用（在合适的选择项前打“☑”）

1.本计划中是否使用了危险物质： ☑ 不使用 ☐ 使用，如使用请填写下面内容

2.详细描述有害物质的名称、来源、剂量、使用方法

名称	类型	来源	使用方法
	☐放射性 ☐致癌物<br>☐重组 DNA ☐危化品<br>☐感染性 ☐病毒载体<br>☐其它（ ）		

3.详细描述对人、动物、环境的危害性，以及采取的个人防护措施和废弃物处理方法

危害性：<br>个人防护措施：<br>废弃物处理方式：

十一. 项目负责人的承诺:

1. 我保证在此提请的陈述是完整的且准确的，本方案中的动物使用没有单纯重复以前的实验。
2. 如果方案被批准，我同意以书面形式告知动物管理与使用委员会（IACUC）任何的紧急情况，且同意在问题解决之前将不会继续实验。
3. 没有提交实验方案的书面修改稿给 IACUC，且经 IACUC 审查、批准之前，我将不会对涉及动物的程序做出较大的改动。
4. 没有 IACUC 和恩施州中心医院动物实验中心主任批准之前，我不会带参观者进入动物设施，不允许他们对动物进行拍照或者摄影。
5. 保证每个从事与动物有关的工作人员接受合适的培训是我的职责。
6. 在收到 IACUC 的批准通知之前，我不会开始本方案中所描述的实验操作。
7. 我保证此计划中如果使用到管制药品或危险物质，均符合国家的相关法规，以及学校的相关规定。
8. 我保证此计划中如果使用到生物材料（包括来自于人和动物），均经过特定检验，没有被实验动物中心以外的啮齿动物污染，不含对人或动物有害的病原微生物。
9. 我将保留一个本实验方案的副本及随后所有的通信资料。
10. 我将允许 IACUC 和主治兽医（AV）有权利查看该项目动物实验记录。在需要时，随时提供实验记录供管理部门审查。

项目负责人（签名）：黄国全　　　　日　期：2025 年 04 月 15 日

十二 科室（或院系）审查意见:

科室（或院系）项目审核意见：

同意开展

科室（或院系）盖章：

分管负责人签字：

日期：2025 年 4 月 15 日
