# Supplementary material for: Mechanistic insights into VSIR-mediated AXL regulation and gastric cancer proliferation
Source: iScience. 2026 Sep 18;29(10):117580. doi: 10.1016/j.isci.2026.117580 (PMC13626921; doi:10.1016/j.isci.2026.117580)
Supplement: Supplementary file 5 [file mmc5.pdf]

293T [HEK-293T]细胞STR鉴定报告

一、 材料处理和检验方法

取适量293T [HEK-293T]细胞(编号PC-H2025121109,1×10^6)使用TIANamp Genomic DNA Kit提取DNA，采用Microreader™21 ID System扩增20个STR位点和性别鉴定位点，使用智阅基因分析仪GenReader 7010对PCR产物进行检测，使用GeneMapper Software6软件（Applied Biosystems）对检测结果进行分析，并与Cellosaurus数据库进行比对。

二、 检测结果

实验中阴性及阳性对照结果均正确。

293T [HEK-293T]细胞株的 STR 位点和 Amelogenin 位点的基因分型结果见附表，分型图谱见附图。

三、 分析说明

293T [HEK-293T]细胞株基因组 DNA 扩增后图谱清晰，分型结果良好。

四、 检验结论

1. 293T [HEK-293T]细胞株 DNA 进行细胞 STR 分型结果显示，细胞株中未发现人类细胞交叉污染。
2. 该细胞株 DNA 分型在细胞库中找到与其细胞分型 97.06%相匹配的细胞株，细胞株名称为 HEK293T。

武汉普诺赛生命科技有限公司 质检专用章

网站: www.procell.com.cn
电话: 400-999-2100
邮箱: techsupport@procell.com.cn
地址: 湖北省武汉市高新大道858号生物医药产业园三期C4栋

附表1：293T [HEK-293T]细胞株的STR位点和Amelogenin位点的基因分型结果。

STR Loci	样品名称：PC-H2025121109	数据库名称：HEK293T
Amelogenin	X	X
CSF1PO	11,12	11,12
D2S1338	19	19
D3S1358	15,16,17	15,16,17
D5S818	8,9	8,9
D7S820	11	11
D8S1179	11,12,14	12,14
D13S317	12	12
D16S539	9,13	9,13
D18S51	17,18	17,18
D19S433	18	18
D21S11	28,30.2	28,30.2
FGA	22,23	23
Penta D	9,10	9,10
Penta E	7,15	7,15
TH01	7,9.3	7,9.3
TPOX	11	11
vWA	16,19	16,19
D6S1043	11	
D12S391	19,21	19,21
D2S441	11,15	11,15
Cellosaurus数据库匹配度97.06%，匹配位点数19（https://www.cellosaurus.org/index.html）		

备注：

1. **根据国际细胞鉴定委员会(ICLAC)制定的细胞 STR 鉴定标准，细胞系的匹配度≥80% 时，认为它们具有相关性，即衍生于共同的祖先细胞；匹配度在 55% 至 80% 之间，需要进一步验证相关性；小于 55%，表明两者不具有相关性。**
2. **图谱有效峰为真实的 PCR 条带；小峰和非特异性条带在计算中忽略不计。**
3. **STR 数据比对结果默认 Cellosaurus，数据来源包括 ATCC、DSMZ、JCRB 等细胞库以及文献和资料记载，数据库入口 https://www.cellosaurus.org/index.html。**

附图1：293T [HEK-293T]（编号PC-H2025121109）细胞STR位点和Amelogenin位点的基因分型结果。

Applied Biosystems
GeneMapper Software 6

20251211

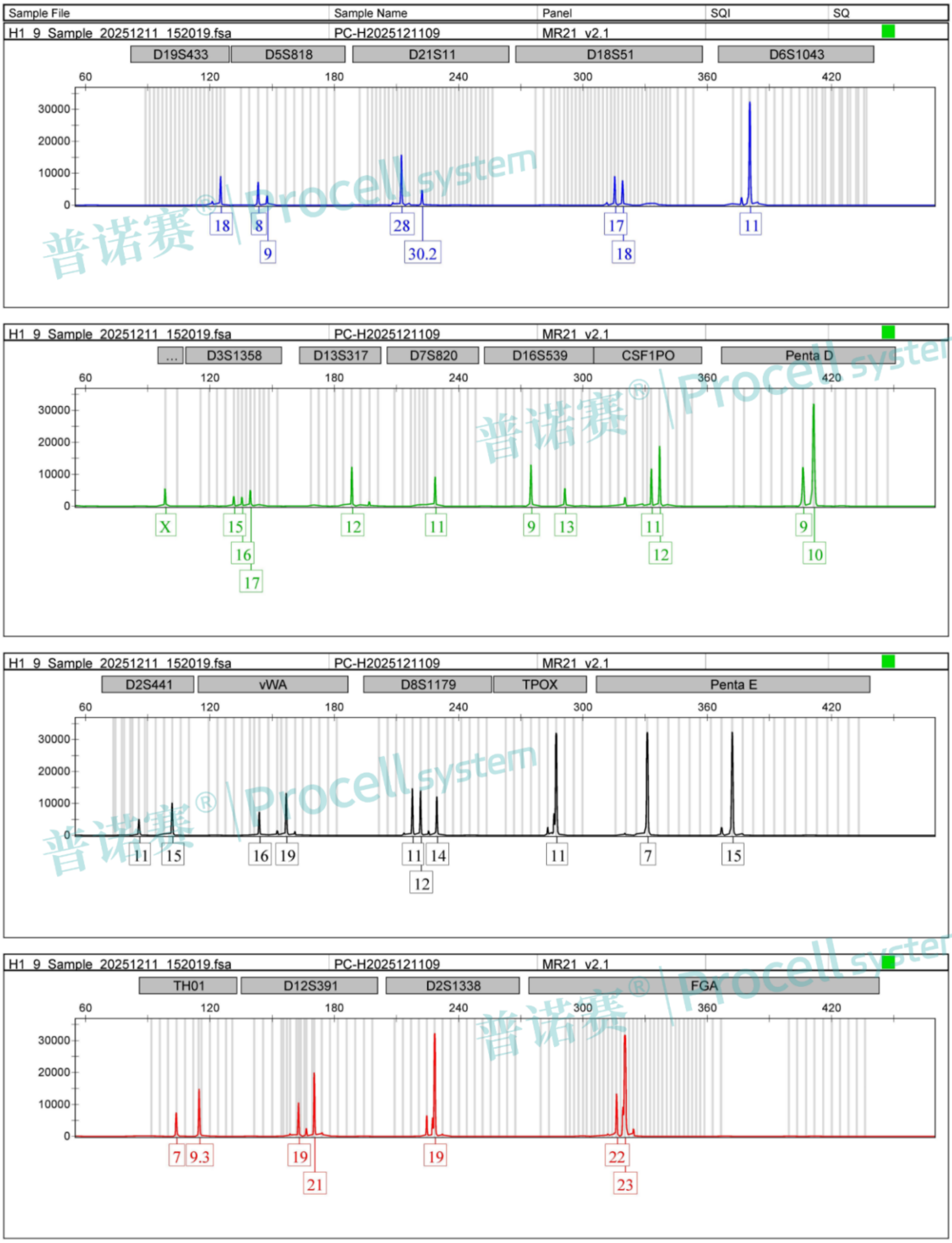

Mon Dec 15,2025 11:18AM, CST
Printed by: gm
Page 1 of 1

网站: www.procell.com.cn
电话: 400-999-2100
邮箱: techsupport@procell.com.cn
地址：湖北省武汉市高新大道858号生物医药产业园三期C4栋
