# Supplementary material for: Mechanistic insights into VSIR-mediated AXL regulation and gastric cancer proliferation
Source: iScience. 2026 Sep 18;29(10):117580. doi: 10.1016/j.isci.2026.117580 (PMC13626921; doi:10.1016/j.isci.2026.117580)
Supplement: Supplementary file 8 [file mmc8.pdf]

GENETIC TESTING BIOTECHNOLOGY

Cell Line Authentication Service
STR Profile Report

**Sample Submitted By:**	Dr. Zhipeng Wang<br>Meisen Cell Technology
**Email Address:**	947638289@qq.com wzppanda@163.com
**Sales Order:**	251028A
**Cell Line Designation:**	THP-1
**Date Sample Received:**	Oct 28th, 2025
**Report Date:**	Oct 29th, 2025

Methodology: Twenty-one short tandem repeat (STR) loci plus the Amelogenin locus were amplified using the commercially available SiFaSTR™ 23 plex Kit. The cell line sample was processed using the ABI Prism® 3130 XL Genetic Analyzer. Data were analyzed using GeneMapper® ID v3.2 software (Applied Biosystems). Appropriate positive and negative controls were run and confirmed for each sample submitted.

Data Interpretation: Cell lines were authenticated using Short Tandem Repeat (STR) analysis as described in 2021 in ANSI Standard (ASN-0002) by the ATCC Standards Development Organization (SDO) and in Jamie L. Almeida et al., Authentication of Human and Mouse Cell Lines by Short Tandem Repeat (STR) DNA Genotype Analysis. Assay Guidance Manual. PMID: 23805434. Bookshelf ID: NBK144066.

GTB™ performs STR Profiling following ISO 9001:2008 and ISO/IEC 17025:2005 quality standards.
There are no warranties with respect to the services or results supplied, express or implied, including, without limitation, any implied warranty of merchantability or fitness for a particular purpose. Genetic Testing Biotechnology (GTB) is not liable for any damages or injuries resulting from receipt and/or improper, inappropriate, negligent or other wrongful use of the test results supplied, and/or from misidentification, misrepresentation, or lack of accuracy of those results. Your exclusive remedy against GTB and those supplying materials used in the services for any losses or damage of any kind whatsoever, whether in contract, tort, or otherwise, shall be, at GTB's option, refund of the fee paid for such service or repeat of the service.

NOTE: According to the recommendations of *IJC* on cell line authentication, the report is valid for 3 years since the issue date.

Technical Questions?
GTB Technical Support
+86-512-67486171
service@jsdna.org
Section 505, Yixin BLD
SIP, Suzhou, 215123
Jiangsu, P.R. China

Ordering Questions?
order@jsdna.org
GTB Corporation
+86-512-62806339
Section 303, Yixin BLD
SIP, Suzhou, 215123
Jiangsu, P.R. China

GENETIC TESTING BIOTECHNOLOGY

Cell Line Authentication Service
STR Profile Report

Sales Order: 251028A

	Test Results for Submitted Sample		ExPASy Reference Database Profile	
**Loci**	**Query Profile:** THP-1		**Database Profile:** THP-1	
Amelogenin	X	Y		
D3S1358	15	17	15	17
D5S818	11	12	11	12
D2S1338	17	18		
TPOX	8	11	8	11
CSF1PO	11	13	11	13
Penta D	10	12		
TH01	8	9.3	8	9.3
vWA	16		16	
D7S820	10		10	
D21S11	30	31.2	30	31.2
Penta E	11	15		
D10S1248	14			
D8S1179	10	14	10	14
D1S1656	16	17		
D18S51	13	14	13	14
D12S391	19			
D6S1043	14			
D19S433	12.2	13		
D16S539	11	12	11	12
D13S317	13		13	
FGA	24	25	24	25

The allele match algorithm compares the 13 core STR loci only, even though alleles from all loci will be reported when available.

Note: Loci highlighted in grey (13 core STR loci) can be made public to verify cell identity. In order to protect the identity of the donor, ***please do not publish*** *the allele calls from all the STR loci tested.*

The sample match is based on the reference data available at the time of comparison.

Explanation of Test Results

Cell lines with ≥80% match are derived from the same donor. Cell lines with between a 70% to 79% match require further profiling for authentication of relatedness. Cell lines with <70% match are very unlikely to be from the same donor.

☐ The submitted sample profile is human, but not a match for any profile in the ExPASy STR database.

☑ The submitted profile is an exact match for the following human cell line(s) in the ExPASy STR database (13 core STR loci): THP-1

☐ The submitted profile is similar to the following ExPASy human cell line(s):

e-Signature Technician: Digitally signed by Xuekun Chen
DN: cn=Xuekun Chen, o=Genetic Testing Biotechnology (Suzhou), ou=DNA Typing Section, email=order@jsdna.org, c=CN
Date: 2025.10.29 14:40:33 +08'00'

e-Signature Reviewer: Digitally signed by Xiankun Zhao
DN: cn=Xiankun Zhao, o=Genetic Testing Biotechnology (Suzhou), ou=Supervision Section, email=service@jsdna.org, c=CN
Date: 2025.10.29 14:46:51 +08'00'

苏州鉴达生物科技有限公司 检验检测 电子专用章

More information

Addendum: Electropherogram for the customer's sample set 1 of 1

Cell Line Authentication Service
STR Profile Report

Applied Biosystems

GeneMapper ID v3.2

251028

Sample Name	Panel	SQO	SQ
THP-1	SifaSTR 23 plex_v7.0		

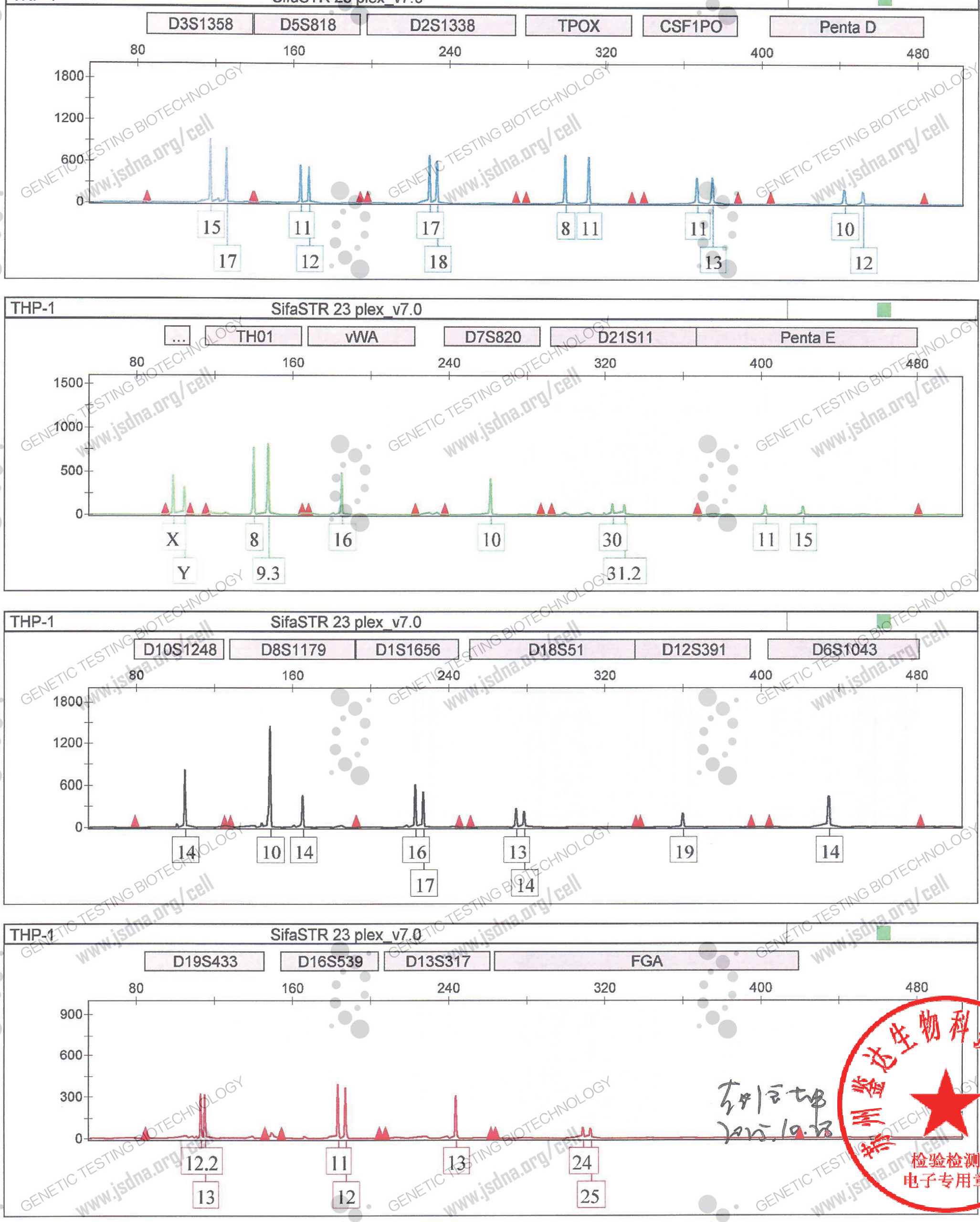

检验检测
电子专用章

Tue Oct 28,2025 03:17PM, CST

Printed by: gmid

Page 1 of 1

GENETIC TESTING BIOTECHNOLOGY www.jsdna.org/cell

Accession	Name	N° Markers	Score	Ameb	CSF1PO	D2S1338	D3S1358	D5S818	D7S820	D8S1179	D13S317	D16S539	D18S51	D19S433	D21S11	FGA	Penta D	Penta E	TH01	TPOX	vWA
NA	Query	NA	NA		11,13		15,17	11,12	10	10,14	13	11,12	13,14		30,31.2	24,25			8,9.3	8,11	16
CVCL_0006 *Best*	THP-1	13	100.00%	X,Y	11,13	17,18	15,17	11,12	10	10,14	13	11,12	13,14	12.2,13	30,31.2	24,25	10,12	11,15	8,9.3	8,11	16
CVCL_0006 *Worst*	THP-1	13	95.83%	X,Y	11,13	17,18	15,17	11,12	10	10,14	8,13	11,12	13,14	13	30,31.2	24,25	10,12	11,15	8,9.3	8,11	16,17
CVCL_E2QQ	THP-1 NF-kappaB-eGFP	13	100.00%	X,Y	11,13		15,17	11,12	10	10,14	13	11,12	13,14		30,31.2	24,25	10,12	11,15	8,9.3	8,11	16
CVCL_XD94	THP-1/IL15	13	95.65%	X	11,13	17,18	15,17	11,12	10	10,14	13	11,12	13,14	12.2,13	30,32.2	24,25		11,15	8,9.3	8,11	16
CVCL_0331 *Best*	Hs 294T	13	68.18%	X	11	17,24	16,17	12	10	14	11,12	11,12	13,17	14,14.2	30	23,24	13	7,10	8,9.3	8,11	16,18
CVCL_0331 *Worst*	Hs 294T	13	66.67%	X	11	17,24	16,17	12	10	13,14	11,12	11,12	13,17	14,14.2	30	23,24	13	7,10	8,9.3	8,11	16,18
CVCL_UN11	3PNF_FiPSsv_PM_2	13	65.22%	X	11,14	17,24	15,16	10,11	10	10,14	8,13	9,12	13,15	12,14.2	30,32.2	21,24			8,9.3	8	16
CVCL_UN12	3PNF_SiPSsv_MM_11	13	65.22%	X	11,14	17,24	15,16	10,11	10	10,14	8,13	9,12	13,15	12,14.2	30,32.2	21,24			8,9.3	8	16
CVCL_2H32	PCL12	13	63.83%	X,Y	11,12	20	16,17	11,13	10	14,15	8,13	11,12	13,14	14,15	30,31.2	20,23	8,9	10,11	9.3	8,11	14,18
CVCL_E9XM	iADE-DM1	13	63.64%	X	11,13	17	16,17	11	10	12,14	11	11	14,15	14,15	30,33.2	21,25			8,9.3	8	16,18
CVCL_2742 *Best*	UM-RC-7	13	63.64%	X,Y	11	17,25	15	12	10	13,14	8,11	11	13,14,19	14,15,16.2	31.2,33.2	24,25	9,12	7,9	8,9	8	16,18
CVCL_2742 *Worst*	UM-RC-7	13	60.47%	X,Y	11	17,25	15	12	10	13,14	8,11	11	14,19	14,15,16.2	31.2,33.2	24,25	9,12	7,9	8,9	8	16,18
